# Evaluation of the Biocompatibility and Osteogenic Properties of Metal Oxide Coatings Applied by Magnetron Sputtering as Potential Biofunctional Surface Modifications for Orthopedic Implants

**DOI:** 10.3390/ma15155240

**Published:** 2022-07-29

**Authors:** Mariana Fernández-Lizárraga, Julieta García-López, Sandra E. Rodil, Rosa María Ribas-Aparicio, Phaedra Silva-Bermudez

**Affiliations:** 1 Posgrado de Doctorado en Ciencias en Biomedicina y Biotecnología Molecular, Escuela Nacional de Ciencias Biológicas, Instituto Politécnico Nacional, Mexico City 11340, Mexico; mfernandezl@ipn.mx; 2 Unidad de Ingeniería de Tejidos, Terapia Celular y Medicina Regenerativa, Instituto Nacional de Rehabilitación Luis Guillermo Ibarra Ibarra, Mexico City 14389, Mexico; garcia.juli2013@gmail.com; 3 Laboratorio de Producción y Control de Biológicos, Departamento de Microbiología, Escuela Nacional de Ciencias Biológicas, Instituto Politécnico Nacional, Mexico City 11340, Mexico; 4 Instituto de Investigaciones en Materiales, Universidad Nacional Autónoma de México, Mexico City 04510, Mexico; srodil@unam.mx

**Keywords:** metal oxide coatings, magnetron sputtering, osteogenesis, mesenchymal stem cells

## Abstract

Biomaterials with adequate properties to direct a biological response are essential for orthopedic and dental implants. The surface properties are responsible for the biological response; thus, coatings with biologically relevant properties such as osteoinduction are exciting options to tailor the surface of different bulk materials. Metal oxide coatings such as TiO_2_, ZrO_2_, Nb_2_O_5_ and Ta_2_O_5_ have been suggested as promising for orthopedic and dental implants. However, a comparative study among them is still missing to select the most promising for bone-growth-related applications. In this work, using magnetron sputtering, TiO_2_, ZrO_2_, Ta_2_O_5_, and Nb_2_O_5_ thin films were deposited on Si (100) substrates. The coatings were characterized by Optical Profilometry, Scanning Electron Microscopy, Energy-Dispersive X-ray Spectroscopy, X-ray Photoelectron Spectroscopy, X-ray Diffraction, Water Contact Angle measurements, and Surface Free Energy calculations. The cell adhesion, viability, proliferation, and differentiation toward the osteoblastic phenotype of mesenchymal stem cells plated on the coatings were measured to define the biological response. Results confirmed that all coatings were biocompatible. However, a more significant number of cells and proliferative cells were observed on Nb_2_O_5_ and Ta_2_O_5_ compared to TiO_2_ and ZrO_2._ Nevertheless, Nb_2_O_5_ and Ta_2_O_5_ seemed to induce cell differentiation toward the osteoblastic phenotype in a longer cell culture time than TiO_2_ and ZrO_2_.

## 1. Introduction

In the orthopedic and dental fields, different efforts have been done to develop improved biomaterials capable of fulfilling the different requirements for their use as temporal osteosynthesis implants to treat bone fractures or critical bone defects [1,2], or as permanent implants such as knee total replacement prosthesis or dental implants [3,4,5,6]. 

For bone-substituting implants, the standard requirements are mechanical resistance and load-bearing capacity, which are associated with the material’s bulk properties [7,8,9]. However, the bone-implant junction (osseointegration), the osteosynthesis performance (osteogenesis capability), the ability to prevent infections, and the corrosion resistance are surface-related properties [4,10,11,12,13]. 

An interesting strategy to tailor the surface properties is the development of biofunctional coatings. In the last few years, increasing interest has been focused on developing coatings to harness the surface properties of different bulk materials and their interaction with the biological media [4,13,14,15,16,17,18]. Titanium oxide coatings with different morphologies, crystalline structures, Ti:O ratios, etc., have been the most evaluated after demonstrating that the native oxide of Ti-based implants is responsible for their biocompatibility (chemical inertness) and excellent osseointegration observed in dental implants [19,20,21,22,23,24,25,26]. However, the extensive use of Ti-based and TiO_2_ materials has raised concerns regarding their low bioactivity, decreasing corrosion resistance in F^−^ or Cl^−^ containing media after long-term use, and the consequent adverse effects of titanium accumulation and its effects on the human body [3,10,27,28,29]. Reports about Ti-associated allergic reactions and hypersensitivity demand research on alternative materials [30,31,32]. 

Other biocompatible transition metal oxides have also shown promising biological properties such as osseointegration, improved cell adhesion and proliferation, decreased inflammatory response and antibacterial properties, along with exhibiting very good corrosion and wear resistance [10,33,34,35,36,37,38,39,40,41,42,43]. However, a significantly smaller number of works have studied the biological response of promising oxides, such as Nb_2_O_5_ and Ta_2_O_5_ [44,45,46,47,48,49]. Another possibility is zirconium oxide, ZrO_2_, which presents mechanical strength, appropriate corrosion resistance and adequate biological response for intraosseous use [10,34,40,50,51,52,53,54]. 

A recent review shows that pure Nb_x_O_y_ and Nb_x_O_y_-containing coatings deposited using dry and/or wet techniques affect the overall characteristics of the underlying bulk materials, where the deposited layers not only address the deficiencies of the biomaterials, such as corrosion resistance but also induce some new properties, e.g., antibacterial activity [55]. For sputtered deposited Nb_x_O_y_ coatings, the improvement in the biological properties is a function of surface topography, wettability, and atomic order [55]. The amorphous atomic order showed superior human fibroblast cell adhesion and antibacterial activity [56], similarly to our previous results for TiO_x_ and ZrO_x_ [57,58]. Moreover, an important finding was the anti-inflammatory properties observed for Nb_x_O_y_-coated metallic substrates, which decreased the toxicity [55].

Yin-Yu et al. [59] compared amorphous and crystalline tantalum oxide coatings deposited by magnetron sputtering, finding that the hydrophilic crystalline β-Ta_2_O_5_ coating presents good biocompatibility for human skin fibroblast cells. In contrast, the amorphous tantalum oxide coating possessed antibacterial properties, in agreement with N. Donkov et al. [60]. Horandghadim et al. [48] showed that the higher the content of Ta_2_O_5_ on hydroxyapatite-Ta_2_O_5_ coatings, the higher the osteoblast-like cell attachment and the bone-like apatite growth. In agreement, F. Wang et al. [47] demonstrated higher cell adhesion and viability, and enhanced calcium deposition and expression of osteogenic genes on Ta_2_O_5_-coated titanium nanotubes compared to the bare nanotubes. In the same trend, Almeida-Alves et al. [44] and H.-L. Huang et al. [61] reported higher MC3T3, human skin fibroblasts, and human osteosarcoma MG-63 cells viability on Ta_2_O_5_ coatings compared to bare Ti. 

More research has been performed on ZrO_2_ as a bulk and coating material [10,34,50]. The use of zirconia in the medical field has expanded over the past twenty years, driven by its advantageous physical, biological, esthetic, and corrosion properties [10,34]. For orthopedic hip replacements, it has superior wear-resistance compared to metals, and the esthetic factor has benefited its use in dentistry for all-ceramic crowns [50]. However, as a bulk material, the risk of catastrophic fracture remains a concern [34]. Concerning ZrO_2_ coatings, M. Peron et al. showed enhanced cell viability for L929 fibroblasts in contact with lixiviates from ZrO_2_-coated Mg alloys compared to TiO_2_-coated and bare Mg alloys’ lixiviates [53,54]. In the same trend, J. Maminskas et al. demonstrated more significant cell adhesion and growth of HGF-1 cells on ZrO_2_ coatings than on bare Ti and Ti alloys [52]. On the other hand, S. Saleem et al. [51] demonstrated a lower friction coefficient and wear rate for ZrO_2_-coated titanium substrates than for the bare substrates.

These ceramic oxides could be applied as coatings with the advantage that they modify the surface properties preserving the bulk properties because they can be deposited over any substrate material that fulfills: (1) the mechanical requirements of the application, either a rigid polymer or a metallic substrate, and (2) the micro roughness requirements for osseointegration [14,18,62]. Coatings can be deposited using techniques such as Atomic Layer Deposition, Electron Beam, sol-gel, etc. In particular, magnetron sputtering is a versatile, clean deposition technique that allows the deposition of conformal and homogeneous coatings, with controlled chemical composition and thickness, at an appropriate deposition rate, and it can be industrially scalable to coat three-dimensional substrates [63,64,65,66]. In the industry, magnetron sputtering has been successfully used to coat a variety of substrates for different applications, e.g., architectural glass for energy efficiency buildings, electronics for the communication industry, or cutting tools for the construction industry [64,67,68,69]. By controlling the deposition parameters such as the power supply, the deposition time, the cathode-anode distance, the magnetron sputtering discharge configuration (planar, cylindrical, rotating, single or multiple blanks, etc.), the sample holder (rotating or static), etc. three dimensional structures of different materials can be conformally and homogeneously coated [64,67,68,70,71].

Surface roughness in the micro range (1–100 µm; substrate roughness) modifies cell proliferation and viability, and osseointegration [58,72,73,74]. Nevertheless, surface nano roughness also modifies the cell response by controlling the protein layer adsorbed on the material surface and, consequently, the extracellular matrix composition [58,75,76,77]. Thus, it is important to deposit the metal oxide coatings on nano-flat substrates such as atomically flat Si (100) wafers for evaluating the biological response to the properties of the oxide coating without interference by the substrate micro roughness.

Despite the different reports evaluating the application of metal oxide coatings for potential application in the biomedical area, to the best of our knowledge, there is no report comparing the biological and osteogenic response under the same experimental conditions. 

In the present work, titanium oxide, zirconium oxide, niobium oxide and tantalum oxide thin films were deposited by magnetron sputtering on Si (100) substrates to study their potential as surface modifications (metal oxide coatings) for orthopedic and dental implants. The physical–chemical properties of the coatings were characterized by optical profilometry, Scanning Electron Microscopy (SEM), Energy-Dispersive X-ray Spectroscopy (EDS), X-ray Photoelectron Spectroscopy (XPS), X-ray Diffraction (XRD), water contact angle measurements (wettability) and Surface Free Energy (SFE) calculations. The biocompatibility and osteogenic properties of the coatings were characterized by using bone marrow-derived mesenchymal stem cells, and studying their adhesion, viability, proliferation, and differentiation toward the osteoblastic phenotype upon culture on the coatings. 

## 2. Materials and Methods

### 2.1. Metal Oxide Coatings Deposition

**Figure 1:**
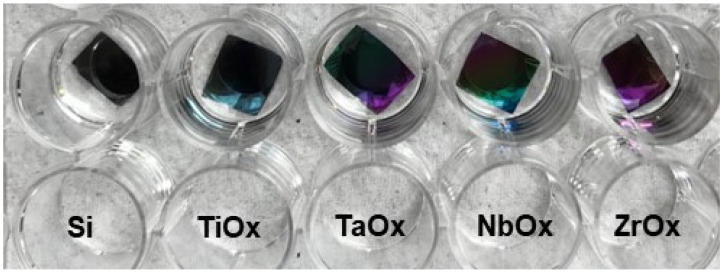
Representative pictures of the macroscopic appearance of Si (100) wafers coated with metal oxide thin films by magnetron sputtering. Coated samples were named as TiOx, TaOx, NbOx, ZrOx, for titanium, tantalum, niobium and zirconium oxide coatings, while Si samples correspond to uncoated Si (100) wafers.

Metal oxide thin films were deposited on Si N/PH (100) wafers (UniSil Corporation, Sta. Clara, CA, USA) and cut into 1 cm^2^ substrates. Atomically flat Si wafers were used as the substrates to examine the influence of the properties of the metal oxide thin films deposited, mainly their roughness and wettability, on the biological response, with no influence of the substrate roughness. Deposition of the metal oxide thin films, titanium oxide (TiOx), tantalum oxide (TaOx), niobium oxide (NbOx), and zirconium oxide (ZrOx), was carried out by magnetron sputtering using 4” in diameter, high purity, metallic targets of either Ti, Ta, Nb or Zr (SCI Engineered Materials, Ohio, MA, USA). Deposition conditions were the same for all coatings deposited; base pressure was below 2 × 10^−6^ Torr, a reactive Ar:O_2_ (80:20) atmosphere was used to a working pressure of 22 mTorr, 200 W incident RF power was applied, and deposition was carried out for 45 min. Figure 1 shows the macroscopic appearance of the coated samples.

Si (100) samples with the middle section covered by permanent marker ink were coated in the same batches as the non-covered, bare Si (100) substrates. After the coating process, ink was removed, exposing the bare Si surface, and creating an appropriate step (bare Si surface- metal oxide-coated Si) to measure the thickness of the metal oxide coatings by profilometry. 

### 2.2. Metal Oxide Coatings Characterization

The morphology of the coated samples and their elemental composition were characterized using a JEOL 7600F Field Emission-Scanning Electron Microscope (FE-SEM) (JEOL USA Inc., Peabody, MA, USA) coupled to EDS, using a voltage of 5.0 kV. For the characterization of the elemental composition by EDS, three different areas (≈10 µm^2^) on two independent samples for each coating group were analyzed, and the average elemental composition (in at.% and wt.%) is presented along with the standard deviation. Elemental and chemical surface composition analyses were performed by X-ray Photoelectron Spectroscopy in a Physical Electronics Versa Probe^TM^ II system with a scanning XPS microprobe (Physical Electronics Inc., Chanhassen, MN, USA), using an Al Kα X-ray source (1486.6 eV and 100 μm beam). To assure the homogeneity and reproducibility of the coatings, different zones on two independent samples per coating group were analyzed. Survey spectra were recorded at 117.4 eV pass energy and high-resolution spectra at 23 eV. Data were analyzed in the Multipack^©^ version 9.6.0.15 software.

Wettability and Surface Free Energy were characterized in an OCA 15EC equipment (DataPhysics Instruments, Filderstadt, Germany) using the Sessil drop contact angle method, and 4 µL drops of distilled water for wettability determinations and 4 µL drops of distilled water, glycerol, isopropanol and dimethyl sulfoxide (DMSO) for surface energy calculations. Contact angles were measured using the SCA_20^®^ software, and the Owens, Wendt, Rabel & Kälble (OWRK) equation was applied to calculate the SFE. For wettability measurements, the stability of the water droplet size over time was measured using the same droplet volume and technique described above. Droplet size and shape were continuously monitored and recorded during the first 5 min immediately after droplet deposition on the coating surface. Images of the droplet at 0, 1, 2, 3, 4 and 5 min after droplet deposition were analyzed. All measurements were done in triplicate.

Crystallinity was characterized by X-ray Diffraction in grazing incidence mode using an Ultima IV diffractometer (Rigaku, Corporation, Tokyo, Japan) with Cu Kα radiation (40 kV, 44 mA). To perform the measurements, the following experimental parameters were set: 0.5° incidence angle, 0.5°/min scanning speed, acquisition step of 0.02°, and 2θ range from 20–70°. TiO_2_ and ZrO_2_ crystalline phases in the TiOx and ZrOx coatings were identified by comparing the X-ray diffraction patterns obtained with the 00-021-1276 and 00-021-1272, and 00-007-0343 PDF cards, correspondingly for TiOx and ZrOx. Crystalline phase identification, average crystal grain size, and crystallinity analysis were performed using the PDXL2^®^ software. Crystal grain size for crystalline samples was calculated by two different methods: the Scherer equation indexing the (1,1,1) and (1,1,−1) diffraction peaks for ZrO_2_ (Baddeleyite), and the (1,1,0) and (2,1,1) diffraction peaks for Rutile and (2,1,1) diffraction peak for Anatase for TiO_2_. The crystal grain size was also calculated by the Williamson–Hall method. Average crystal grain sizes are presented. 

Coatings thickness, surface topography imaging, and average roughness (Sa) were measured in a Zygo Nexview^TM^ optical profilometer (Zygo Corporation, Middlefield, CT, USA) using the Mx^TM^ software (version 6.4.0.21, Zygo Corporation, Middlefield, CT, USA) for data analysis. 

### 2.3. Cells

Human Bone Marrow-derived Mesenchymal Stem Cells (BM-MSC; PCS-500-012, ATCC^®^, Manassas, VA, USA) were used for all biological experiments. Cells were expanded in a Mesenchymal Stem Cell Basal Medium (PCS-500-030; ATCC^®^, Manassas, VA USA) supplemented with Mesenchymal Stem Cell Growth Kit (PCS-500-041; ATCC^®^, Manassas, VA, USA), Penicillin-Streptomycin-Amphotericin B solution (PCS-999-002; ATCC^®^, Manassas, VA, USA) and phenol red (PCS-999-001; ATCC^®^, Manassas, VA, USA), in the concentrations recommended by the cells’ supplier. Cells were used within a maximum of 9 passages, as indicated by the quality control certificate. Cells were incubated at 37 °C and 5% CO_2_, and the medium was changed every three days.

Even though BM-MSC in passage 1 were obtained with a quality control certificate from ATCC^®^, their expression of positive and negative mesenchymal stem cells markers and their differentiation capacity towards chondrogenic, adipogenic and osteogenic lineages were evaluated at passage 6 by flow cytometry and histological staining, respectively. Expression of positive CD90, CD105, CD73, and negative CD45, CD34 and HLA MSC surface markers from BM-MSC were measured in a flow cytometer FACSCalibur^TM^ (Beckton Dickinson, Franklin Lakes, NJ, USA) using the Cell Quest Pro software (V.5.2.1., Beckton Dickinson, Franklin Lakes, NJ, USA) for data acquisition and the FlowJo^TM^ software for data analysis (V.10.8.1., Beckton Dickinson, Franklin Lakes, NJ, USA). BM-MSC cells were independently incubated in specific (osteogenic, adipogenic, and chondrogenic) differentiation-inducing culture media, and cells differentiation toward the specific phenotype (depending on the specific differentiation-inducing media used) was corroborated by histological staining; that is, alizarin red, alcian blue and oil red staining were used to identify osteoblastic, chondrogenic and adipogenic differentiation, respectively. Details of methods and results for BM-MSC characterization are presented in Supplementary Materials: “Bone-marrow derived mesenchymal stem cells characterization”. 

### 2.4. Cell Attachment and Viability of BM-MSC on the Metal Oxide Coatings

Cell adhesion and viability upon culture on the metal oxide coatings were qualitatively and quantitatively evaluated. The coatings samples were sterilized with UV light on both sides for 20 min each side. Then, samples were placed in 24-well tissue culture plates and BM-MSC were drop-seeded on the samples at a density of $4\times 10^{3}$ cells/cm^2^. To allow the cells’ attachment to the sample’s surface, cells-drop-seeded samples were incubated at 37 °C and 5% CO_2_ for 90 min. Then, DMEM/F-12 supplemented with 10% *v*/*v* Fetal Bovine Serum (FBS; Gibco, Thermo Fisher Scientific, Waltham, MA, USA) and 1% *v*/*v* antibiotic-antimycotic was added to the wells up to a 400 µL volume, and culture plates were placed back into the incubator. Independent samples for each coating group were cultured for 3, 7 and 14 days, changing the culture medium every three days. Three different culture time points were chosen to evaluate cell adhesion, viability, and morphology over time up to the cell culture time span when cell differentiation is expected to occur: 14 days. 

To evaluate the adhesion and morphology of the BM-MSC at the specific culture time points, independent cell-cultured samples were rinsed twice with PBS 1X and fixed overnight with 2.5% glutaraldehyde (Sigma Aldrich, Waltham, MA, USA). Then, samples were rinsed again with PBS 1X and progressively dehydrated in 20%, 40%, 60%, 80% and 100% ethanol solutions (JT Baker^TM^, Radnor, PE, USA). Samples were allowed to dry at room temperature (RT), and images were acquired in a Zygo Nexview^TM^ optical profilometer (Zygo Corporation, Middlefield, CT, USA) using the Mx^TM^ software (version 6.4.0.21, Zygo Corporation, Middlefield, CT, USA). 

To evaluate the cells’ viability at the specific culture time points, the LIVE/DEAD^TM^ Viability/Cytotoxicity Kit for mammalian cells (Invitrogen^®^, Massachusetts, USA) was used on independent cell-cultured samples following the kit manufacturer’s instructions. After incubation with the LIVE/DEAD^TM^ kit (Invitrogen^®^, Waltham, MA, USA), samples were rinsed twice with PBS and immediately visualized by Fluorescence Microscopy in an Axio Observer microscope (Carl Zeiss, Jena, Germany) using the AxioVision^®^ software (Version Rel 4.8.2, Carl Zeiss, Jena, Germany) to acquire the images. Cell viability was also quantitatively evaluated on independent cells-cultured samples using the Alamar Blue^TM^ cell viability kit (Thermo Fisher Scientific, Waltham MA, USA, USA) according to the kit’s manufacturer protocol. Briefly, at the specific culture time points established, the culture medium was removed and replaced in the darkness by 10:1 DMEM/F-12:Alamar Blue^TM^ solution. Then, samples were placed back in the incubator for 2 h, and finally, 100 µL aliquots of the supernatants were collected, and the absorbance was read at 570 nm in a Synergy-HTX multi-mode reader spectrophotometer (Bio Tek Instruments, Winooski, VT, USA). A calibration curve was obtained by seeding different known numbers of cells on independent wells in standard tissue culture plates (TCP). After 24 h of cell culture, cell viability was evaluated following the same procedure as described above for the Alamar Blue assay. The calibration curve calculated the number of viable cells from absorbance readings. 

For all techniques described, but Alamar Blue assay, BM-MSC were also cultured on uncoated Si samples for comparison purposes. Cell viability positive controls (Ctrl+) corresponded to cells cultured on TCP under standard culture conditions. All experiments were carried out in triplicate. 

### 2.5. Proliferation of BM-MSC on the Metal Oxide Coatings

Procedures used for cell seeding and culture on the coatings were the same as described in Section 2.4, but cell seeding density was $3.5\times 10^{3}$ cells/cm^2^ and two different culture times were handled (3 and 5 days) in order to avoid reaching a high cell density on the surface, and the consequent inhibition of cell proliferation due to cell–cell contact phenomenon. After the culture time-points indicated, cell proliferation was evaluated using the Cell Proliferation ELISA, BrdU (colorimetric) kit (Roche, Basilea, Switzerland), following the kit’s manufacturer instructions and setting the incubation time for BrdU labeling solution at 4 h. After removing the labeling medium, samples were dried using a hair dryer for 15 min and stored at 4 °C to continue with the Cell Proliferation ELISA BrdU kit protocol the next day. All experiments were carried out by triplicate.

### 2.6. Evaluation of the Osteogenic Properties of the Metal Oxide Coatings

An evaluation of cell differentiation toward the osteogenic lineage upon culture on the metal oxide coatings was performed by Immunofluorescence (IF) and ELISA assays.

For IF assays, cells were seeded on the coated samples under the same procedure as described in Section 2.5. Three different osteogenic markers were chosen to be evaluated: RUNX2, osteopontin (OP), and osteocalcin (OC). For RUNX2, OP, and OC expression characterization, independent cell-seeded samples were evaluated after 5, 7 and 14 days of cell culture, respectively. Different days of culture were chosen for the evaluation of the different markers due to their expression occurring at different stages of the cell differentiation process. The culture medium was DMEM/F-12 supplemented with 2% *v*/*v* FBS and 1% *v*/*v* antibiotic-antimycotic, and incubation conditions were 37 °C and 5% CO_2_. Human osteoblasts and fibroblasts cells cultured in TCP were used as controls of positive and negative markers expression, respectively; Supplementary information: *Immunofluorescence assays controls*. BM-MSC cells culture in TCP under standard culture conditions were used as comparative controls of the natural level of markers expression from BM-MSC. 

For RUNX2 and OP, the following IF protocol was followed: after corresponding culture time, the culture medium was removed, samples were rinsed twice with a Phosphate Buffer Saline solution (PBS; Gibco Thermo Fisher Scientific, Waltham, MA, USA), incubated in methanol (Sigma–Aldrich, Michigan, USA) for 5 min at RT, rinsed three times with PBS, incubated in Triton 100 × 0.2% (Sigma-Aldrich, St. Louis, MO, USA) in PBS for 30 min, rinsed three times with PBS, and incubated in a PBS-Tween-BSA-FBS-glycine blocking solution (PBS, Tween 0.1% (USB Corporation, Cleveland, OH, USA), BSA 1% (Sigma-Aldrich, St. Louis, MO, USA), FBS 10%, and glycine 0.3 M (MP Biomedicals, Irvine, CA, USA)) for 1 h at RT in a humid chamber. Then, a primary antibody was added, either anti-RUNX2 ab76956 (Abcam, Cambridge, UK) at 1:50 dilution in PBS-1% BSA solution or Anti-Osteopontin ab8448 (Abcam, Cambridge, UK) at 1:100 dilution in PBS-1% BSA solution and samples were incubated for 1 h at RT. After incubation, samples were rinsed five times with the 0.2% PBST (PBS-Tween) solution and incubated for 1 h with the corresponding secondary antibody, either Anti-Mouse ab150105, Alexa Fluor^®^ 488 (Abcam, Cambridge, UK) at 1:1000 dilution in PBS-1% BSA solution for RUNX2 or Donkey Anti-Rabbit Alexa Fluor^®^ 488 (Abcam, Cambridge, UK) at 1:2000 dilution in the PBS-1% BSA solution (Abcam, Cambridge, UK) for OP. Finally, samples were rinsed five times with PBST 0.2%, cells nuclei were counterstained with Hoechst 33342 (Invitrogen^TM^, Carlsbad, CA, USA) at a 1:1000 dilution in PBS, and samples were placed on a slide with a mounting medium for microscope observation. IF protocol for OC was as follows: after 14 days of cells culture on the coating samples, the culture medium was removed, samples were rinsed twice with PBS, fixed with PFA 4% for 10 min at RT, and rinsed three times with PBS 1X. Then, samples were incubated in a blocking solution (PBST 0.2% and BSA 8%) for 30 min, rinsed three times with PBS 1X, and incubated for 1 h with the primary antibody anti-Osteocalcin ab93876 (Abcam, Cambridge, UK) at a 1:200 dilution in blocking solution at RT in a humid chamber. Then, samples were rinsed five times with PBST 0.2%, and incubated for 1 h at RT with the secondary antibody Donkey Anti-Rabbit Alexa Fluor^®^ 488 (Abcam, Cambridge, UK) at a 1:2000 dilution in PBS-1% BSA solution. Finally, samples were rinsed five times with PBST 0.2%, cell nuclei were counterstained with Hoescht solution, and samples were placed on microscope slides with mounting medium. Mounted samples were visualized by Fluorescence Microscopy using an Axio.Observer microscope (Carl Zeiss, Jena, Germany) and the AxioVision^®^ software, using 100× magnification. Experiments were carried out in triplicate.

The expression of osteogenic markers was quantified using ELISA assays. BM-MSC were seeded on coated samples and cultured for 14 days in DMEM-F/12 medium supplemented with 10% FBS and 1% antibiotic-antimycotic at 37 °C and 5% CO_2_. The culture medium was replaced every three days. BM-MSC cultured on TCP with Mesenchymal Stem Cells basal medium were used as controls of negative expression of osteogenic markers. After culture time, the culture medium was replaced by fresh DMEM-F/12 with no FBS, and samples were further incubated for 24 h. Then, supernatants were collected and used to quantify marker expression following the ELISA kits manufacturer’s instructions. ELISA kits used were: Human Osteocalcin Simple Step ELISA^®^ kit ab270202 (Abcam, Cambridge, UK); Osteoprotegerin Human ELISA kit ab100617 (Abcam, Cambridge, UK); and Osteopontin Human ELISA kit ab100618 (Abcam, Cambridge, UK). All assays were normalized to the number of viable cells using the Alamar Blue assay for quantification of the number of viable cells on the samples. Experiments were carried out in triplicate. Alkaline Phosphatase Diethanolamine Detection kit AP0100 (Sigma–Aldrich, St. Louis, MO, USA) was also used according to the manufacturer’s instructions to quantify ALP expression at 7 and 14 days for cell culture.

### 2.7. Statistical Analysis

All data are presented as the mean ± standard error using three independent experiments per variable. Statistical significance was determined by a one-way ANOVA analysis with a Dunnett’s post-hoc test, using the Graph Pad Prism 9.1.0 software (Graph Pad by Dotmatics, Boston, MA, USA). Values of *p* < 0.05 were considered statistically significant.

## 3. Results

### 3.1. Metal Oxides Coatings Characterization

**Figure 2:**
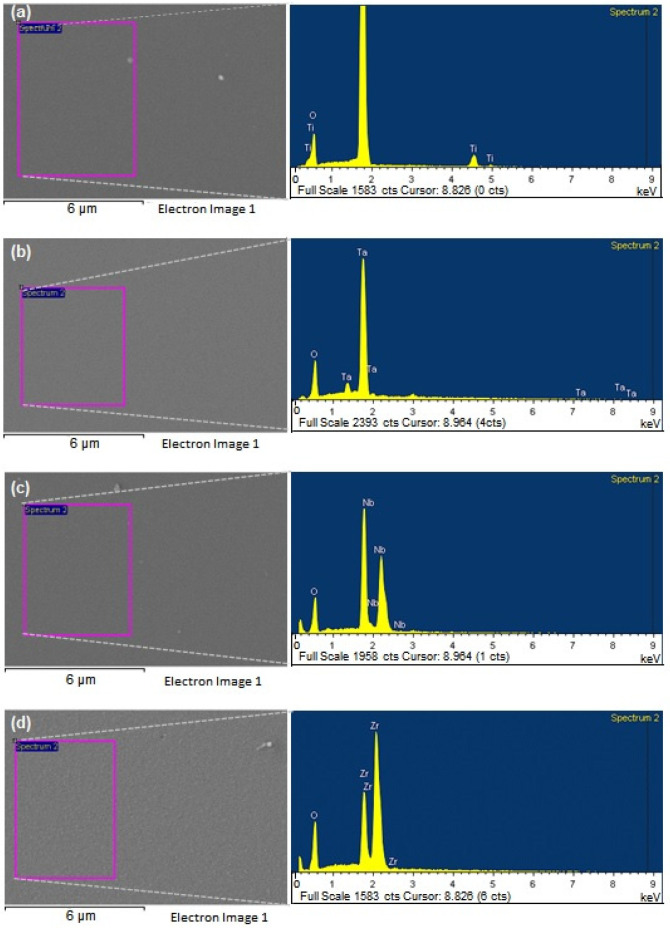
Representative SEM images of the areas analyzed (pink rectangles marked on the micrographs) for EDS elemental composition studies and of the corresponding EDS spectra obtained for the (**a**) TiOx; (**b**) TaOx; (**c**) NbOx; and (**d**) ZrOx coated samples.

Representative SEM micrographs exhibiting the microscopic morphology of the samples are shown in Figure 2, along with their elemental composition spectra as obtained by EDS. From micrographs in Figure 2, it is possible to observe a smooth morphology for all metal oxide coatings studied, which was later corroborated by a profilometry roughness analysis. Chemical elements identified for each coating are shown in the EDS spectra (Figure 2), exhibiting the expected elements depending on the oxide coating and no signs of trace contamination within the EDS detection limit. Table 1 shows the atomic and weight percentage of the identified chemical elements as calculated from EDS.

Table 2 includes the results from the optical profilometry images (topography), arithmetical average roughness (Sa), and thickness of the metal oxide coatings. Average roughness is expressed as the average of the Sa measured on the whole surface of three different coated samples per metal oxide group of study. As Table 2 shows, all Sa values were smaller than 1 nm, indicating conformal deposition of metal oxide films on the atomically flat substrates (Si (100) wafers) used. The coatings thickness values represent the average of at least eight measurements performed on three independent samples per metal oxide group of study. In all cases, the average thickness was larger than 50 nm. The thinnest coating was TiOx (54.53 nm), while thickness increased, respectively, for NbOx, ZrOx, and TaOx, which corresponded to the thickest coating (346.49 nm). The thickness is not expected to influence the early biological response toward the metal oxide coatings, but latter phenomena such as corrosion or biodegradation might be sensitive to the thickness and homogeneity of the coatings. 

**Figure 3:**
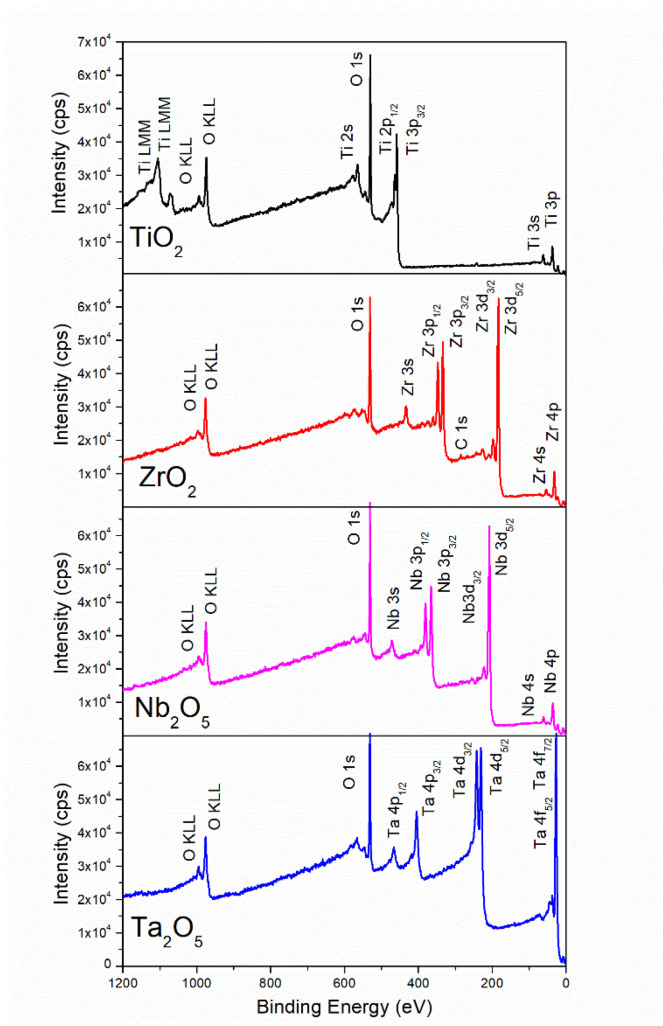
Example low resolution XPS spectra of TiO_X_ (TiO_2_), ZrO_X_ (ZrO_2_), NbO_X_ (Nb_2_O_5_) and TaO_X_ (Ta_2_O_5_).

Representative survey XPS spectra of the metal oxide coatings after Ar^+^ ion cleaning are shown in Figure 3. In all cases, the spectra evidenced photoelectron peaks corresponding only to O and to the metal in the oxide: Ti, Ta, Nb, or Zr, corroborating no trace elements contamination in the coatings. A minor C component was observed for ZrOx due to a shorter Ar^+^ ion cleaning performed on this coating to avoid sub-stoichiometric oxides formation, which occurred upon longer Ar^+^ ion cleaning; deformation of the Zr photoelectron peaks was clearly observed upon long-time Ar^+^ ion cleaning. There were no signals of the Si substrate for any of the metal oxide coatings (different regions in at least two different samples per oxide group were analyzed), indicating that the coatings homogeneously and uniformly cover the substrate. Even the thinnest coating deposited, TiOx, did not show any evidence of photoelectron peaks from the Si substrate. Average metal:oxygen ratio for the coatings, as calculated from XPS spectra, were 0.48 ± 0.02 for TiOx; 0.40 ± 0.01 for TaOx; 0.40 ± 0.03 for NbOx and 0.48 ± 0.08 for ZrOx. Evidencing the formation of nearly stoichiometric oxides for all coatings deposited; that is, TiO_2_ for TiOx, Ta_2_O_5_ for TaOx, Nb_2_O_5_ for NbOx, and ZrO_2_ for ZrOx. Metal:oxygen ratios calculated by XPS differed from those as calculated from the at.% elemental composition obtained by EDS. Smaller metal:oxygen ratios (around 0.25) are obtained from EDS data. The differences in the measurement techniques can address this. Firstly, the volume of analysis in EDS is deeper, and some detected oxygen might be associated with the native SiO_2_ layer. Secondly, during EDS measurements, samples are in vacuum but are not Ar^+^ ion cleaned before the measurements; thus, traces of organic compounds (containing oxygen) and oxygen from the atmosphere might remain adsorbed on the surface, increasing the amount of oxygen in the coatings’ surface, and consequently decreasing the calculated metal:oxygen ratio from EDS data, in comparison to that obtained from XPS measurements. 

**Figure 4:**
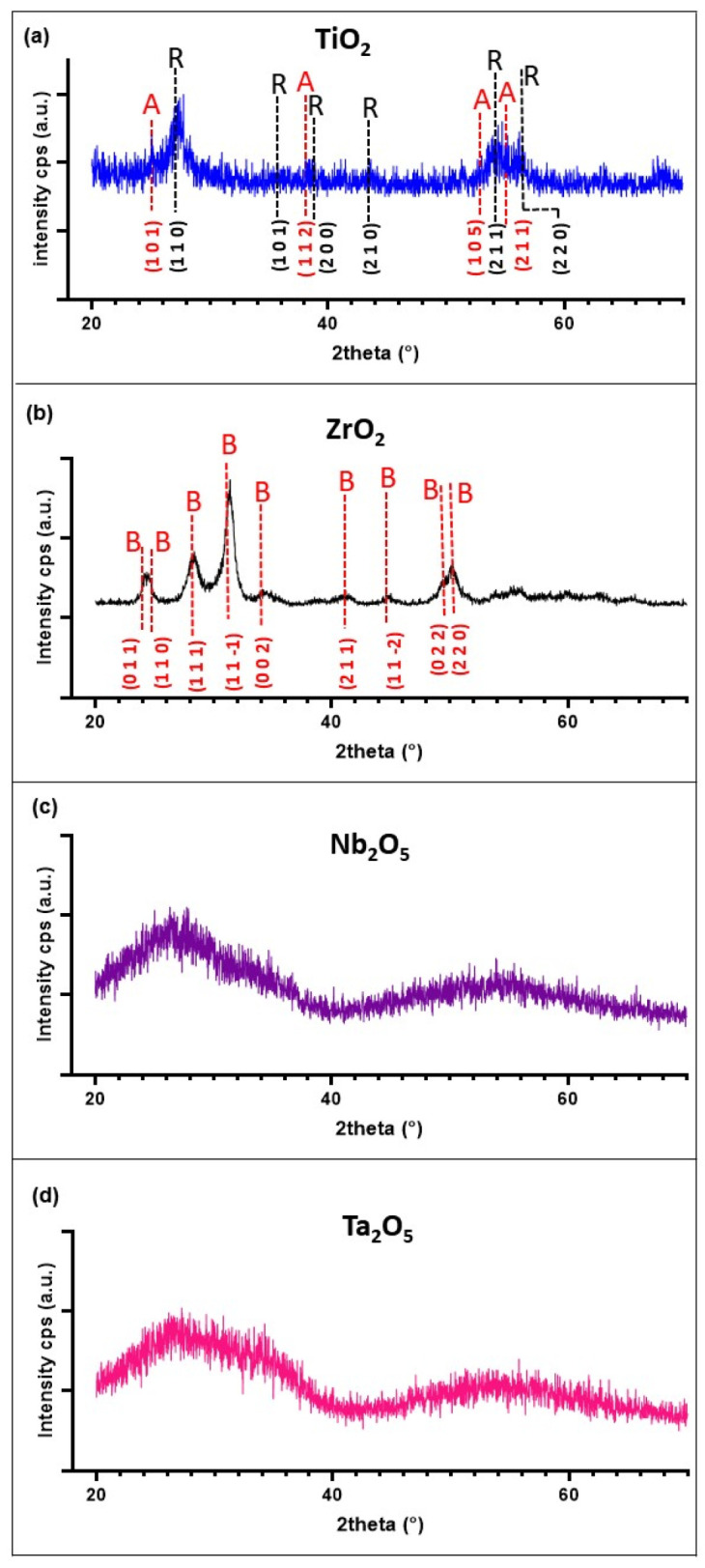
Representative grazing incidence XRD patterns of (**a**) TiOx, (**b**) ZrOx, (**c**) NbOx, and (**d**) TaOx coatings. In Figure 3a, the A and R stand for the expected XRD pattern for Anatase and Rutile crystalline phases of TiO_2_, respectively. In Figure 3b, the B stands for the expected XRD pattern for the Baddeleyite crystalline phase of ZrO_2_.

The GI-XRD diffraction patterns shown in Figure 4 indicate that both TaOx and NbOx coatings were amorphous. Two broad peaks were observed for these two coatings; however, the average crystallite size calculated from these peaks would be smaller than 1 nm. Consequently, a significantly large number of crystal grains frontiers would exist within the material, causing significant disorder in it so that it can be confidently considered as X-ray amorphous. On the other hand, TiOx and ZrOx showed a nanocrystalline structure with an average crystallite size of 5 ± 1 and 7 ± 2 nm for TiOx and ZrOx, respectively. ZrOx nanocrystalline structure corresponded to the monoclinic phase, baddeleyite, while the TiOx XRD pattern indicated a mixture of the tetragonal crystalline phases, anatase (50.5%) and rutile (49.5%). ZrOx can be considered crystalline with no amorphous part since there was no observable amorphous contribution evidenced by the quite linear background observed. On the other hand, a slight non-linearity can be observed in the background of the XRD spectra for TiOx. Calculating the amorphous-crystalline contributions for this coating, an 87% crystallinity was obtained. 

The wettability (WCA) and SFE of the coatings were evaluated by contact angle measurements. The stability of the water droplets over time on the coating’s surface is shown in Table 3, exhibiting the image footage at initial water droplet deposition, and at 1, 2, 3, 4, and 5 min after droplet deposition. From Table 3, a decrease in droplet size with time can be observed for all coatings. Nevertheless, variations in droplet shape, and consequently contact angle, were not relevant; thus, a decrease in droplet size can be mainly attributed to evaporation effects due to water angle measurements performed in an open chamber. From results obtained from water droplet stability measurements over time, it was established to measure WCA at 30 s after droplet deposition on the coating’s surface. In the case of the liquids used for calculating SFE, the same time span was considered except for isopropanol, where contact angles were calculated at 15 s after droplet deposition. Isopropanol has a higher vapor pressure and, consequently, a higher evaporation rate [78], drastically decreasing the droplet’s time to remain stable. 

Table 4 shows the average WCA values, as well as the total SFE (γ_d/*p*_) and the analysis of the polar (γ_p_) and dispersive (γ_d_) components of the total SFE. According to the results obtained for WCA, all coatings can be conventionally considered as having a hydrophilic nature, exhibiting WCA < 90°, being ZrOx the one closest to the hydrophilic–hydrophobic limit. Total SFE was similar among all coatings, ≈24 mN/m with a larger dispersive component compared to the polar component. Total SFE increased from TaOx and ZrOx, to TiOx and NbOx, presenting similar total SFE. γ_p_/(γ_p_ +γ_d_) is known as the polarity factor and indicates the polar fraction of the surface, where TaOx exhibited the highest polar character and decreased to NbOx and ZrOx that exhibited similar polar characters, and to TiOx, which presented the smallest polar character. 

### 3.2. BM-MSC Characterization

BM-MSC cells in passage 6 were characterized by evaluating their expression of the main positive and negative mesenchymal stem cell surface markers, as well as evaluating their capacity to differentiate towards the chondrogenic, adipogenic, and osteogenic lineages, upon incubation with specific differentiation-inducing media; according to the International Society of Cell Therapy. BM-MSC cells exhibited more than 95% positive expression of surface mesenchymal stem markers CD90, CD105, and CD73, and less than 2% of expression of the hematopoietic stem cells markers CD45, CD34, and HLA; Supplementary Figure S1. BM-MSC cells exhibited a fibroblast-like cell morphology (passage 8) incubated in supplemented mesenchymal stem cell basal medium (Supplementary Figure S2a). After 14 days of culture in a chondrogenic-inducing medium, BM-MSC cells were positively stained with alcian blue, indicating synthesis of proteoglycans and corroborating chondrogenic differentiation (Supplementary Figure S2b). After 17 days of incubation in an osteogenic-inducing differentiation medium, BM-MSC were positively stained with alizarin red, exhibiting the formation of calcium deposits, indicative of osteogenic differentiation (Supplementary Figure S2c). Finally, after 12 days of incubation in an adipogenic-inducing differentiation medium, BM-MSC showed lipid vesicles positively stained with oil red dye, demonstrating adipogenic differentiation (Supplementary Figure S2d). It was confirmed that BM-MSC cells used in the present study preserved the main characteristics of mesenchymal stem cells up to passage 8.

### 3.3. Viability of BM-MSC Cultured on the Surface of the Coatings

**Figure 5:**
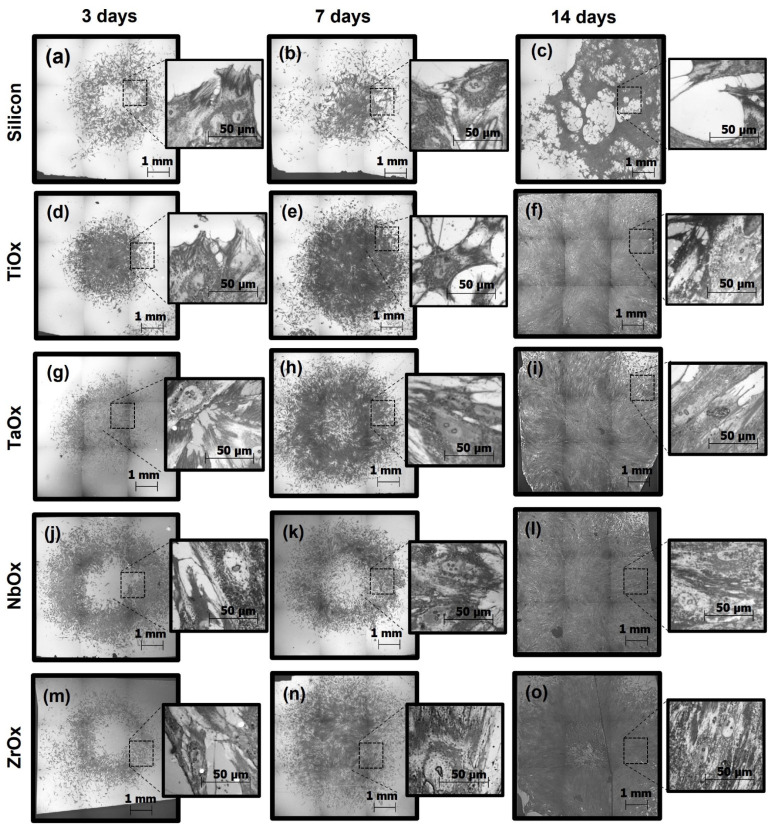
Representative micrographs, obtained by an optical profilometer, of BM-MSC cell cultured on uncoated Si (**a**–**c**) and coated, TiOx (**d**–**f**), TaOx (**g**–**i**), NbOx (**j**–**l**), and ZrOx (**m**–**o**), samples. Three incubation times were handled, 3, 7, and 14 days. Micrographs were acquired at two different magnifications, 5× for main micrographs and 50× for insert magnifications.

Cell adhesion, morphology, and viability of BM-MSC cultured on uncoated Si surfaces and the coatings were evaluated at 3, 7 and 14 days of culture. Figure 5 shows representative micrographs of the cell cultures (after cells fixation) acquired in an optical profilometer. At 5× magnification, main micrographs, the entire sample surface can be observed, which allows for visually evaluating the density and distribution of cells over the entire surface of the samples, and with culture time. These images allow qualitative evaluation of cell proliferation (increment of cell density on the surface) and adhesion over the whole sample surface. Higher magnification images, 50×, allow for precise observation of cells morphology. In the case of BM-MSC cultured on the uncoated Si substrates (Figure 5a–c), the cells initially adhered to the surface, displaying a similar morphology to those of the cells cultured on the coatings. However, at 7 days of culture, fewer cells are observed on Si compared to the coatings, and by day 14, cells detachment from Si was clearly observed. On the coatings, at 3 days of culture (Figure 5d,g,j,m), cells were well-adhered to the surfaces displaying the expected fibroblast-like morphology of well-adhered MSC. For the coatings, as culture time increased, cell density raised to cover the entire surface at 14 days of culture, indicating cell proliferation. At 7 days of culture (Figure 5e,h,k,n), cells exhibited similar extended morphologies on all the coatings, signaling appropriate adhesion to the surface. At 14 days of culture, cells cultured on ZrOx (Figure 5o) exhibited a slight cell detachment phenomenon occurring in the center of the cell monolayer, probably due to a significantly higher cell density in that area. Cells appeared to be well adhered to the ZrOx surface, other than the center; however, cell morphology seemed to be more elongated and in a more compact array compared to cells cultured on TiOx, TaOx, or NbOx.

**Figure 6:**
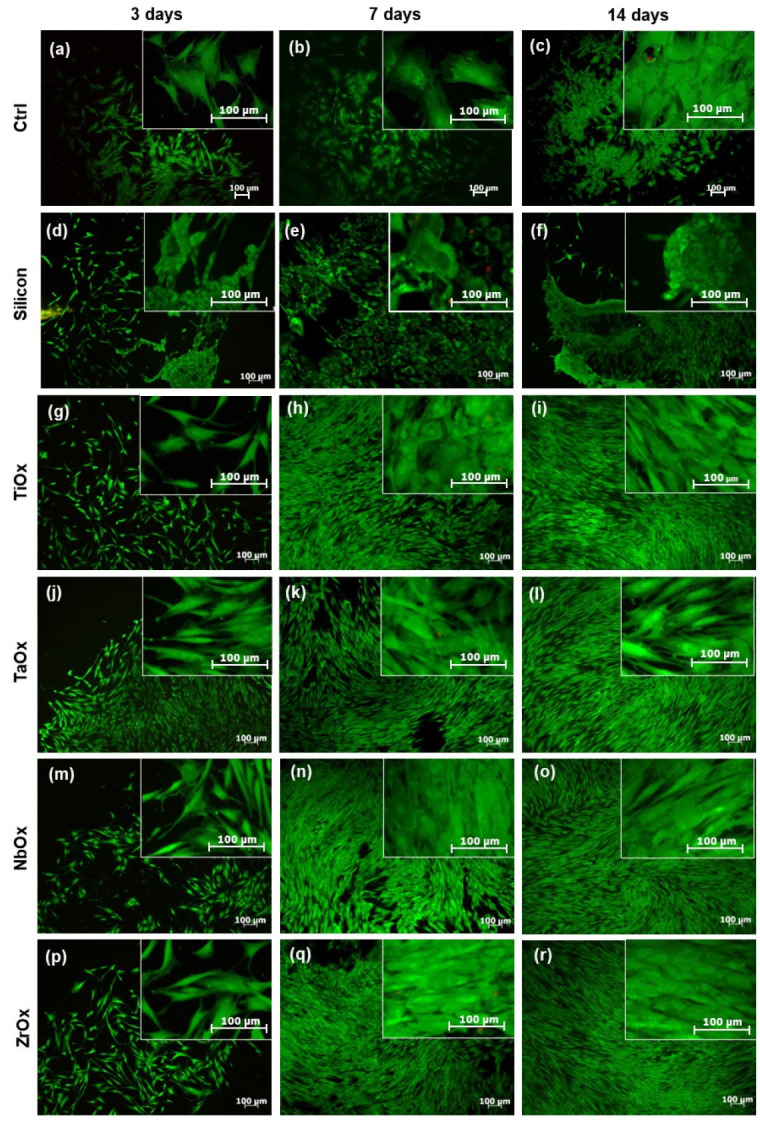
Qualitative evaluation of BM-MSC viability at 3, 7, and 14 days of culture on uncoated Si (**d**–**f**) and TiOx (**g**–**i**), TaOx (**j**–**l**), NbOx (**m**–**o**), and ZrOx (**p**–**r**) coated samples. Ctrl (**a**–**c**) corresponds to BM-MSC culture on the surface of standard tissue culture plates. Micrographs were acquired at two different magnifications, 5× and 20×. The fluorescent LIVE/DEAD^TM^ Viability/Cytotoxicity Kit (Invitrogen^®^, Waltham, MA, USA) for mammalian cells was used to mark viable (green) and dead (red nuclei) cells.

Figure 6 presents a qualitative evaluation of cell viability by the LIVE/DEAD^®^ fluorescence assay. Viable cells are marked in green, and dead cells are marked in red. Upon cell culture on uncoated Si samples, cells were viable; however, they presented a more rounded morphology and became easily detached from the surface compared to the cells cultured on the coatings. In addition, a smaller number of viable cells and a higher number of dead cells were observed at all culture times on Si compared to the coatings. Upon culture on the coatings, cells were viable at all culture times studied, showing just a few dead cells. In concordance with what was observed by optical profilometry (Figure 5), LIVE/DEAD assay showed that cell density increased on the coatings surfaces with culture time, and corroborated that cells were not only well-adhered to the coatings and increasing their population with culture time, but also were viable at all culture times on the coatings.

**Figure 7:**
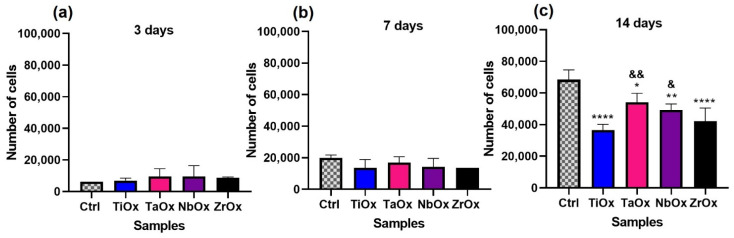
Quantitative evaluation of BM-MSC viability cultured on the coatings at (**a**) 3 days, (**b**) 7days, (**c**) 14 days of culture. Thermo Fisher Alamar Blue^TM^ cell viability reagent kit was used. Ctrl corresponds to BM-MSC cultured on the surface of standard tissue culture plates. Data are presented as the mean ± standard error using three independent cultures per variable and analyzed by one-way ANOVA using a Dunnett’s post hoc test. * *p* ≤ 0.05 vs. Ctrl; ** *p* ≤ 0.001 vs. Ctrl; **** *p* ≤ 0.0001; & *p* ≤ 0.05 vs. TiOx; && *p* ≤ 0.01 vs. TiOx.

Cell viability was also quantitatively assessed using the Alamar Blue reagent. For this assay, BM-MSC cultured on uncoated Si were not considered due to the negative results observed for this surface in the qualitative evaluations of cell adhesion and viability (Figure 5 and Figure 6). According to Figure 7a, the number of viable cells at 3 days of culture was similar between all coatings and the Ctrl; no significant differences were found in the statistical analysis. The Ctrl corresponded to BM-MSC cultured on the surface of standard TCP. After 7 days of culture (Figure 7b), the number of viable cells increased for all coated surfaces and the Ctrl; the number of viable cells was comparable for all coatings studied and the Ctrl with no significant differences. An increase in the number of cells with culture days indirectly indicates cell proliferation. At 14 days of culture (Figure 7c), the number of viable cells increased on all coatings compared to 7 days of culture; however, the number of viable cells on the coatings was significantly smaller than that on the Ctrl. It is essential to mention that in no case the number of viable cells on the coatings was smaller than 50% of the number of viable cells on the Ctrl. TaOx and NbOx showed the highest number of viable cells (there were no significant differences between them) among the coatings studied, followed by ZrOx, and finally TiOx, which showed the smallest number of viable cells; the number of viable cells was not significantly different between TiOx and ZrOx.

### 3.4. Proliferation of BM-MSC Cultured on the Coatings

**Figure 8:**
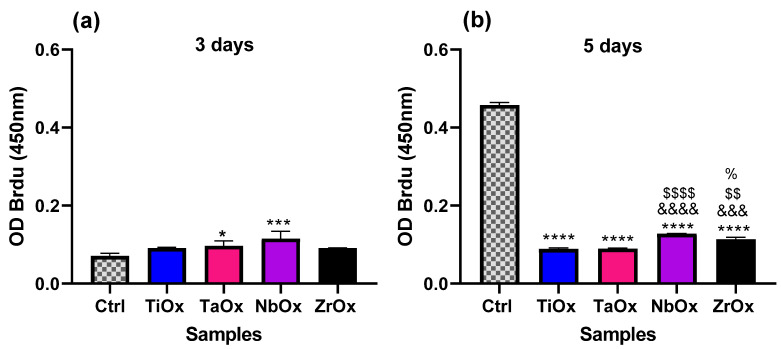
Evaluation of cell proliferation for BM-MSC cultured on the coatings studied. (**a**) 3 days and (**b**) 5 days of culture. The Roche Cell Proliferation ELISA, BrdU (colorimetric) kit was used. Ctrl corresponds to BM-MSC cultured on standard TCP. Data are presented as the mean ± standard error using three independent cultures per variable and analyzed by one-way ANOVA using a Dunnett’s post hoc test. * *p* ≤ 0.05 vs. Ctrl; *** *p* ≤ 0.005 and **** *p* ≤ 0.0005; &&& *p* ≤ 0.001 vs. TiOx; &&&& *p* ≤ 0.0001 vs. TiOx; $$ *p* ≤ 0.01 vs. TaOx; $$$$ *p* ≤ 0.001 vs. TaOx; % *p* ≤ 0.05 vs. NbOx.

Cell proliferation upon culture on the coatings was evaluated using the Cell Proliferation ELISA, BrdU (colorimetric) kit at 3 and 5 days of BM-MSC culture. Ctrl corresponds to BM-MSC cultured on the surface of standard TCP. Figure 8a shows that at 3 days of culture, a higher percentage of the viable cells on TaOx and NbOx were in a proliferative state (BrdU assay absorbance read) since, according to Figure 7, the total number of cells was similar to the Ctrl up to 7 days of culture. However, there was no significant difference in the percentage of viable cells in a proliferative state among the different coatings studied. At 5 days of culture (Figure 8b), cells cultured on all the coatings exhibited a significantly smaller percentage of viable cells in a proliferative state compared to the Ctrl. The observed decrease in cell proliferation rate at 5 days compared to the control might be that cells attached to the coating’s surface have already initiated the differentiation process, as observed in the next section. Among the cells cultured on the coatings, NbOx showed the largest percentage of viable cells in a proliferative state at 5 days of culture, and this difference was significant against TiOx, TaOx, and ZrOx. After NbOx, the second-largest percentage of viable cells in a proliferative state among the coatings was observed on ZrOx, which exhibited a significantly larger percentage of viable cells in a proliferative state in comparison to TiOx and TaOx. There was no significant difference in the percentage of viable cells in a proliferative state between TiOx and TaOx.

### 3.5. Osteogenic Properties of the Coatings

**Figure 9:**
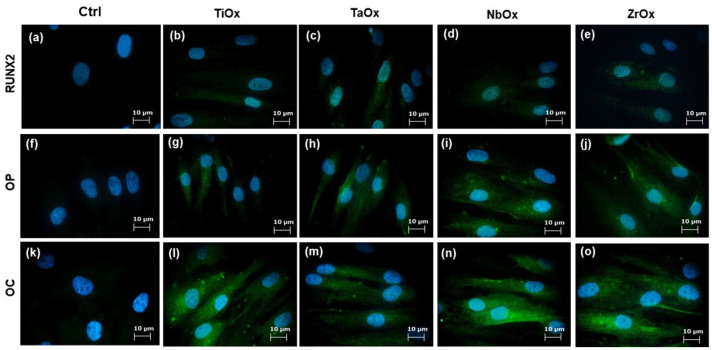
RUNX2 (**a**–**e**), OP (**f**–**j**) and OC (**k**–**o**) cellular expression characterized by IF assays in BM-MSC cultured on the coatings. In green, positive expression of the corresponding osteogenic marker is observed, while in blue, cell nuclei stained with Hoechst are observed. Ctrl corresponds to cells cultured on standard tissue culture plates.

Cell differentiation toward the osteogenic lineage was evaluated by qualitative IF and quantitative ELISA assays. Figure 9 displays representative micrographs of the expression of RUNX2, OP, and OC osteogenic markers from BM-MSC cultured on the coatings. RUNX2 is a transcription factor; thus, secondary antibody labeling is observed in and around the cell nucleus (Figure 9b–e). OP (Figure 9g–j) and OC proteins are expressed mainly in the cell cytoplasm (Figure 9l–o). Figure 9a,f,k correspond to BM-MSC cultured on TCP (Ctrl), which allowed for the evaluation of the basal expression of the osteogenic markers studied from BM-MSC. It has been described that FBS used in the culture medium might have induced a certain level of osteogenic differentiation [79]. Then, for the present evaluation, cells were incubated with DMEM/F-12 supplemented with 2% FBS; thus, the potential of osteogenic differentiation observed can be mainly attributed to the external stimulus studied, in this case, the contact with the coatings. According to the results observed in Figure 9, there was a positive expression of RUNX2, OP and OC in all the coatings studied. This positive expression of osteogenic differentiation markers can be confidently correlated to the effect of the coatings since the Ctrl presented a minimal expression of OP and OC, and no expression of RUNX2. Cellular controls for the IF assays are shown in the Supplementary Information (Supplementary Figures S3 and S4).

**Figure 10:**
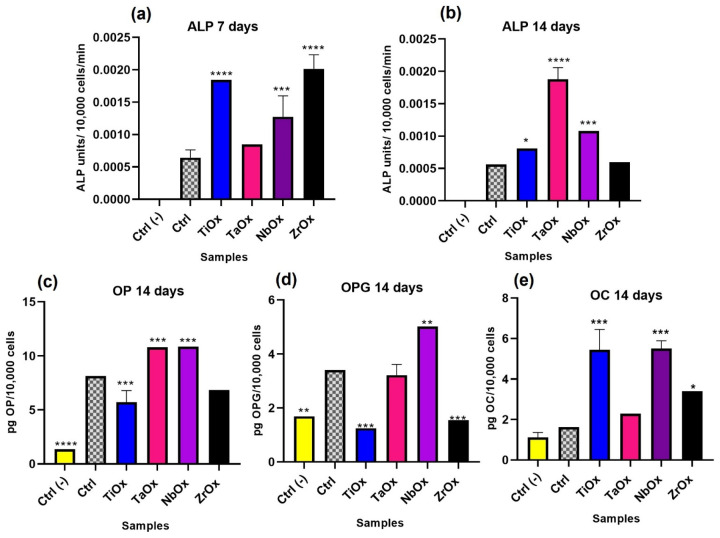
Quantification of proteins involved in osteogenic differentiation. Quantification of ALP activity, at (**a**) 7 days, and (**b**) 14 days of cells culture. Quantification of (**c**) osteopontin (OP) expression, (**d**) osteoprotegerin (OPG), and (**e**) osteocalcin (OC) expression after 14 days of cells culture. Ctrl- and Ctrl correspond to BM-MSC cultured on TCP surfaces with supplemented Mesenchymal Stem Basal Medium (Ctrl−), or DMEM/F-12 supplemented with 10% *v*/*v* FBS (Ctrl). Data are presented as the mean ± standard error using three independent cultures per variable and analyzed by one-way ANOVA using a Dunnett’s post hoc test. * *p* ≤ 0.05 vs. Ctrl; ** *p* ≤ 0.01 vs. Ctrl; *** *p* ≤ 0.001; and **** *p* ≤ 0.0001 vs. Ctrl vs Ctrl.

Osteogenic differentiation was also quantitatively evaluated. Figure 10 shows the quantification of some proteins involved in the differentiation process of MSC toward the osteogenic lineage. For protein quantification, supernatants were collected, and ELISA commercial kits were used; a colorimetric assay kit was used for quantification of ALP activity. Two independent comparison cell controls were set, consisting of BM-MSC cultured on TCP with a supplemented Mesenchymal Stem Basal Medium (Ctrl-) and BM-MSC cultured on TCP with DMEM/F-12 supplemented with 10% *v*/*v* FBS (Ctrl). So, it was possible to compare the proteins intrinsically expressed by mesenchymal stem cells (Ctrl-) with the basal expression of osteogenic markers expressed by mesenchymal stem cells incubated in FBS-supplemented medium (Ctrl), which might per se induced a certain level of osteogenic differentiation. 

Regarding the results for ALP activity at 7 days of cell culture (Figure 10a), there was no ALP activity detected in the Ctrl-, and the largest ALP activity was observed on TiOx and ZrOx, which presented similar ALP activity between them, but significantly larger ALP activity compared to the Ctrl. ALP activity on NbOx was smaller than that TiOx and ZrOx but still significantly larger than the Ctrl. ALP activity on TaOx was similar to that of the Ctrl. At 14 days of cell culture (Figure 10b), there was no ALP activity detected in the Ctrl-, while ALP activity was detected on all coatings, with TaOx, presenting the largest value. The ALP activity decreased from TaOx to NbOx, TiOx and ZrOx; NbOx and TiOx presented a higher ALP than the Ctrl, while ZrOx exhibited a similar ALP activity to that of the Ctrl. 

In the case of OP (Figure 10c), protein expression in the Ctrl- was significantly smaller than that for the Ctrl and the coatings. TaOx and NbOx expressed the highest amount of OP, exhibiting significantly larger OP expression than cells cultured on TiOx, ZrOx or the Ctrl. Cells cultured on ZrOx and TiOx expressed a significantly smaller amount of OP than the Ctrl. For OPG (Figure 10d), the most extensive protein expression was observed for cells cultured on NbOx, where OPG expression was significantly larger compared to all the other coatings studied and to the Ctrl. OPG expression from cells cultured on TaOx was similar to that observed for the Ctrl. OPG expression on ZrOx and TiOX was significantly smaller than that observed for the Ctrl and similar to the OPG expression observed for the Ctrl-. Finally, for OC (Figure 10e), protein expression was similar between the Ctrl- and the Ctrl. OC expression was significantly larger on all the coatings studied in comparison to the Ctrl. The most significant OC expression among the coatings corresponded to cells cultured on NbOx and TiOx, which expressed similar levels of OC between them, followed by OC expression from cells cultured on ZrOx and TaOx. Cells culture on TaOx expressed the smallest amount of OC among the different coatings studied; however, OC expression on TaOx was still significantly larger in comparison to the Ctrl. 

## 4. Discussion

Several studies have searched for appropriate surfaces to promote the osseointegration process in orthopedic implants. It is known that surface modifications allow harnessing of the biological response that may significantly reduce implant failure or patient rejection. Although appropriate biological responses being triggered by certain surface modifications are known, there is still a long way to go in developing biomaterials with the optimal surface features that ensure a successful osseointegration process [80]. Many techniques have been described for obtaining positive surface modifications. Some of them add structures on the substrate surface (coatings), while others modify the existing ones (acid etching, sandblasting, etc.) [80]. In this work, surfaces coated with metal oxide thin films (Ti, Ta, Nb and Zr oxides) were generated by magnetron sputtering. This technique allows the deposition of coatings on various materials that meet the desirable mechanical properties for orthopedic implants and allows tailored surface chemistry by varying the deposition conditions [67,68,69,70,71]. Therefore, this technique represents a promising proposal for in vitro and in vivo investigation, and for its application at an industrial level in the generation of orthopedic implants. 

To elucidate the correlations of the biological response observed toward the properties of the coating (TiO_2_, Ta_2_O_5_, Nb_2_O_5,_ ZrO_2_), an appropriate physical and chemical characterization of the oxide coatings is very important. The present work characterized the coatings by SEM, EDS, XPS, XRD, wettability, and SFE calculations. The EDS and XPS analysis (Figure 2 and Figure 3) corroborated that the deposited oxide coatings only contained the expected chemical elements, and no traces of other elements were observed. Thus, the cellular response presented in this work can be precisely correlated to the corresponding chemical and elemental composition of the metal oxide coatings and the physical properties emerging from this. 

The difficulty in modifying a particular property without altering others has caused the heterogeneity observed in published results, making it challenging to reach a consensus about the most significant surface properties a biomaterial must meet for developing orthopedic implants [58]. In this sense, to evaluate the effect of the chemical composition on the biocompatibility and differentiation of MSC cells, four metal oxide coatings were deposited on atomically flat Si (100) substrates. Coatings deposition on Si (100) substrates ruled out the influence of roughness and wettability in the biological response, as both could directly affect cell adhesion and proliferation [58,81,82,83]. Wettability due to variations in the surface roughness can directly affect cell adhesion and proliferation [58,81,82,83]. Meanwhile, it has been reported that roughness values between 0.1 and 100 µm favor cell adhesion (specifically in the range of 10–30 µm) [84], while cell adhesion decreased on rougher surfaces (≥100 µm) [58]. At the nanoscale (1–100 nm), roughness indirectly affects cell adhesion by controlling the adsorbed protein layer on the surface [75,76,77,85]. Results in Figure 5 and SFE data suggested that the excellent adhesion and proliferation of the BM-MSC on the coatings are related to the surface chemistry, the rougher surface, or the more hydrophobic character (protein adsorption related) of the coatings since, on the silicon surface (Figure 5b,c), cells did not remain adhered after 7 days of culture. 

Regarding the coatings thickness values obtained (Table 2), all the coatings were thicker than 50 nm, being TiO_2_ the thinnest coating (54.5 nm). Coatings thickness can be correlated to corrosion rates and coating adhesion strength, which are desirable characteristics in an in vivo system. Generally, the thinner the coating, the greater its adhesion strength to the substrate, and the poorer its corrosion protection [86]. In addition, detachment of the coatings might cause the accumulation of debris in the surrounding tissue and consequently cause inflammation and exacerbate immunological responses [32]. Nevertheless, some reports relate the thickness of the coatings with the enhancement of osteogenic properties [87], and the increasing bone attachment to the implant in an in vivo system [88] but always related to another surface feature influenced by coatings thickness, such as surface zeta-potential and porous surface structure. For the evaluations carried out in this work, the thicknesses of the different coatings are not expected to directly affect the biological response as long as the coatings homogeneously cover the substrate surface. Thus, it was important to corroborate that covering of the substrate surface by the metal oxide coatings was complete and homogeneous, a characteristic corroborated by the replicated XPS and EDS measurements on different areas of different coated samples. According to our results, the thickness of the coatings does not seem to have a direct relationship with osteogenic differentiation, since both TiO_2_ and ZrO_2_ (Figure 10) presented a faster osteogenic induction than the other coatings, with TiO_2_ being the thinnest coating and ZrO_2_ one of the thickest. 

Although the differences were not too significant, cell numbers after 14 days of incubation were larger on the amorphous Ta_2_O_5_ and Nb_2_O_5_ surfaces compared to the nanocrystalline TiO_2_ and ZrO_2_ (Figure 7) coatings. In some cases, it has been reported that crystalline structures favor cell adhesion and proliferation; in the case of TiO_2_ it has been described that the anatase phase increases cell adhesion [89,90]. However, the native TiO_2_ surface has been reported as amorphous [91], and in previous works, we showed that cell adhesion and proliferation were promoted on the amorphous TiO_2_ [58] and ZrO_2_ [50] coatings in comparison with their crystalline counterpart. 

Other properties also crucial in cellular adhesion are wettability and surface-free energy. It is known that cells preferentially adhere to hydrophilic surfaces, while proteins tend to adsorb on hydrophobic surfaces efficiently [92]. Nevertheless, on hydrophilic surfaces, protein adsorption depends on the electrostatic interactions, and thus, pH variations in the physiological environment might improve protein adsorption by causing proteins to easily undergo conformational changes that might change their positive/negative charge facilitating their interaction with hydrophilic surfaces; depending on the surface charge [93]. According to the present wettability results (Table 4), hydrophilic oxide coatings seemed to favor cell adhesion and proliferation. All the coatings studied presented a hydrophilic nature, being ZrO_2_ the closest to the hydrophilic–hydrophobic limit. Of the coatings studied, Nb_2_O_5_ presented the highest hydrophilic character, and on this metal oxide, cells covered the largest surface area upon seeding (Figure 5j), and cells proliferation in the first days of incubation (3 and 5 days) was favored (Figure 8). These results are consistent with those reported in previous studies, where a higher cell proliferation was observed at 3 days of incubation on Nb_2_O_5_ compared to TiO_2_ [94]. 

Surface-free energy is involved in protein adsorption and cell adhesion. Proteins are known to more readily adhere to hydrophobic surfaces, but it has been described that on hydrophilic surfaces, the polar component of the surface free energy is directly correlated with the amount of protein adsorbed. In this way, even if the surface is hydrophilic, if its polar component is considerable within the value of the total surface free energy, adsorption of proteins will be favored. Cell adhesion and proliferation are enhanced with increasing SFE, where the polar component has an important role. As the SFE polar component increases, cell adhesion is favored due to easily established bonds between the carboxyl and hydroxyl groups present on the hydrated surface and the lipids and ions in the cell membrane [95]. Table 4, shows the SFE values of the coatings and the polarity factor (γp/(γp +γd)). Ta_2_O_5_ and Nb_2_O_5_ presented the highest contribution of the polar component to the SFE. In the viability assay at 3 days of incubation (Figure 7a), more viable cells were observed on Ta_2_O_5_ and Nb_2_O_5_, in comparison to the other oxides, which can be addressed to the fact that these two oxides have the largest value of the polarity factor. At 7 days of incubation (Figure 7b), a higher number of viable cells was observed on Ta_2_O_5_ compared to the other oxides. All the coatings generated in the present study can be expected to favor cell adhesion and proliferation, being hydrophilic. However, according to the coatings’ surface energy values (dispersive and polar component), it can be hypothesized that, although Ta_2_O_5_ presented a hydrophilic nature, its higher polar component of the surface free energy might have favored appropriate adsorption of proteins, and the consequent appropriate biological response observed [93]. 

When evaluating the viability of BM-MSC cultured on the coatings (Figure 6 and Figure 7), in all cases, cells remained viable up to 14 days of culture, with Ta_2_O_5_ being the coating with the most significant number of viable cells at day 14, followed by Nb_2_O_5_. On the other hand, Nb_2_O_5,_ presented a larger number of proliferative cells at 14 days of cell culture, which had also been previously reported [94]. It is essential to notice that cell proliferation decreases as cell differentiation starts; thus, decreased cell number observed on TiO_2_, ZrO_2_ and on Nb_2_O_5_ at 14 days of cell culture might be also correlated with a faster differentiation of BM-MSC cells occurring on TiO_2_, ZrO_2_, Ta_2_O_5_ and finally Nb_2_O_5_

It is not only desirable that a biomaterial intended to be used in orthopedic implants allows cell adhesion and proliferation, but it is also desirable that it favors the whole osseointegration process, for example, by improving osteoinduction. Figure 9 shows that all the coatings induced a positive expression of the evaluated osteogenic markers (RUNX2, OP, OC, and ALP), which qualitatively demonstrated that on all the four coatings studied, BM-MSC were differentiating towards the osteogenic lineage. Figure 10 shows the quantification of some proteins that participate at different stages of the osteogenesis (osteoinduction) process, which helps us to infer how the differentiation process took place on each of the coatings studied. RUNX2 and ALP are early osteogenic differentiation markers, while OP, OPG, and OC are considered late differentiation markers. RUNX2 is a transcription factor that regulates the differentiation of MSC toward preosteoblasts and regulates the expression of latter osteogenic markers [96]. ALP is a glycoprotein secreted by preosteoblasts and osteoblasts, and it is directly involved in mineralization, in addition to the fact that during osteogenesis, the presence of this enzyme is indicative of a differentiation process of MSC toward osteoblasts [79,97]. OP is an extracellular matrix protein secreted by mature osteoblasts and is involved in various processes, including bone mineralization [98,99]. It has been described that OP is directly involved in improving the osteogenesis process during osseointegration in dental implants [100]. OPG is a protein found in various organs, mainly bone and it is known to be released by osteoblasts, and function as an antagonist of osteclastogenesis [101]. Finally, OC is the most abundant non-collagenous protein in mature bone, and osteoblasts specifically synthesize it; although it has been mainly related to bone formation, several processes in which it could be involved have been also described [102]. According to the protein quantification results in the present study (Figure 10), after 7 days of BM-MSC culture, ZrO_2_ and TiO_2_ exerted the highest ALP activity, indicating that on these two coatings, the induction of osteogenesis might have started earlier than on Ta_2_O_5_ and Nb_2_O_5_. However, by 14 days of culture Ta_2_O_5_ was the one presenting the highest ALP activity, as on this coating the differentiation process started later than in the other coatings. When comparing ZrO_2_ and TiO_2_, only TiO_2_ was the one that presented the highest amount of OC at 14 days of cultures. Thus, probably ZrO_2_ stopped, or was in a later state of, differentiation by 14 days of culture; ALP activity on ZrO_2_ at 7 days of culture was the largest. In Figure 10a, it is observed that Nb_2_O_5_ presented a higher ALP activity with respect to Ta_2_O_5_, at 14 days of incubation, it presented one of the highest expressions of OP, OPG and OC. Finally, Ta_2_O_5_ at 14 days of incubation presented the highest activity of ALP and OP. For OPG it had a lower expression than Nb_2_O_5_, while for OC, Ta_2_O_5_ was the coating with the lowest expression. In this way, it is possible to propose that the induction of osteogenesis was faster in ZrO_2_, followed by TiO_2_, then Nb_2_O_5_ and finally Ta_2_O_5._ This osteogenic differentiation process is also supported by the viability assay, in which at 14 days of incubation (Figure 7c), a lower number of cells on TiO_2_ and ZrO_2_, followed by Nb_2_O_5_ and Ta_2_O_5_ was observed. It has been reported that after 9 days, cell line changes phenotype from proliferative to differentiative [103], process in which a decrease in proliferation occurs to allow differentiation.

Although TiO_2_ continues to be one of the best options for manufacturing orthopedic implants, reports warn about its toxicity [30]. In most cases, these problems are related to the damage in the oxide layer due to corrosion, causing the release of particles that lead to peri-implantitis [32]. According to the present results, ZrO_2_ represents a potential alternative to TiO_2_, displaying ZrO_2_ a similar osteoinduction process to that of TiO_2_; nevertheless, further studies on the mechanisms and timewise proceeding of the differentiation process on these oxides are needed_._ Different studies have reported characteristics of ZrO_2_ that can be comparable with those of TiO_2_. Among the most relevant features, it was reported a higher adhesion of MG63 cells and higher ALP activity at 4 days cultured on ZrO_2_ films compared to TiO_2_ films [104], in addition ZrO_2_has a higher corrosion resistance due to its higher electrochemical stability and surface integrity, which significantly reduces toxicity problems [54]. It has also been described that zirconia layers inhibit bacterial adhesion, which is a very desirable characteristic [105].

## 5. Conclusions

This study shows that cell adhesion, proliferation, and osteogenic differentiation can be promoted by the four metal oxide coatings evaluated: TiO_2_, which can be considered the goal standard to compare, ZrO_2_, Ta_2_O_5_, and Nb_2_O_5_. Small but statistically significant differences were observed in their biological responses, suggesting ZrO_2_ as an alternative substitute for TiO_2_, with the advantage of a faster differentiation process. ZrO_2_ presented the larger water contact (close to 90°) and nano crystalline structure, but intermediate SFE, polarity factor, and thickness among the oxides. Thus, the first two parameters seem to be more critical for osteogenic differentiation.

From our analysis, it can also be observed that the most polar surface (polarity factor of 34%), Ta_2_O_5_, promoted a large number of attached cells, which might be relevant for cell culture applications.

To date, few studies have studied various metal oxides under comparable experimental conditions as possible candidates for potential orthopedic implant applications. Therefore, the results presented in this work represent a starting point for knowing and improving the surface properties of metal oxides for orthopedic applications.

## Figures and Tables

**Table 1:** Coatings elemental composition as obtained from EDS analysis.

Coating	Element	Weight %	Atomic %
TiOx	O	57.8 ± 0.5	80.4 ± 0.3
	Ti	42.2 ± 0.5	19.6 ± 0.3
TaOx	O	17.5 ± 0.3	70.5 ± 0.5
	Ta	82.6 ± 0.3	29.5 ± 0.5
NbOx	O	31.7 ± 0.2	72.9 ± 0.2
	Nb	68.3 ± 0.2	27.1 ± 0.2
ZrOx	O	26.2 ± 0.2	66.9 ± 0.2
	Zr	73.8 ± 0.2	33.1 ± 0.2

**Table 2:** Roughness, representative images of the surface topography, and thickness of the metal oxide coatings.

Coating Property	TiOx	TaOx	NbOx	ZrOx
Sa * (nm)	0.5 ± 0.1 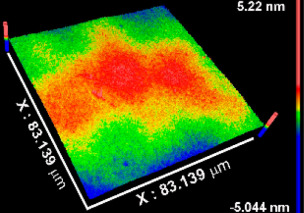	0.4 ± 0.2 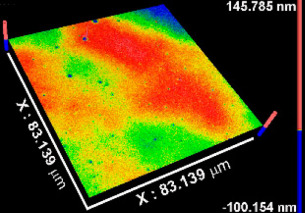	0.8 ± 0.1 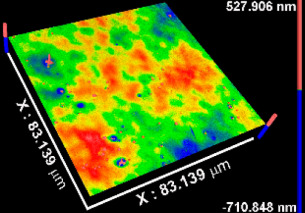	0.3 ± 0.1 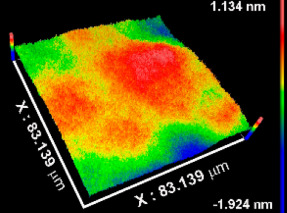
Thickness (nm)	54.5 ± 7.3	346.5 ± 31.5	175.9 ± 15	227.7 ± 18.5

* Sa, arithmetical mean of the roughness.

**Table 3:** Droplet size stability over time for water contact angle measurements.

Sample	Initial Measurement (0 min)	1 min	2 min	3 min	4 min	5 min
Si	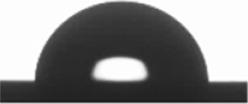	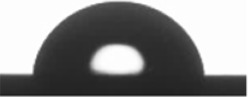	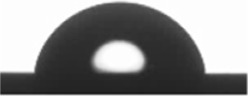	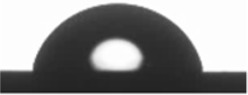	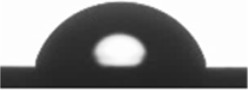	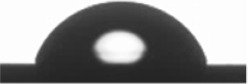
TiOx	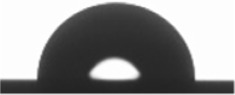	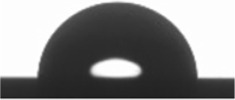	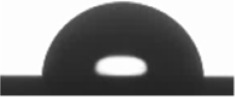	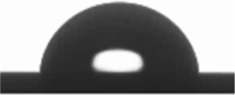	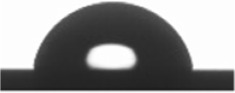	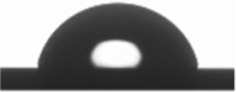
TaOx	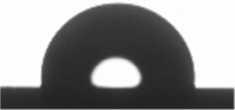	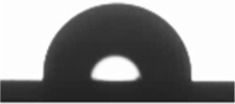	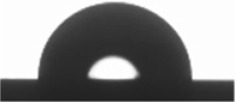	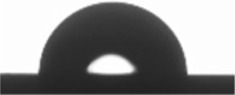	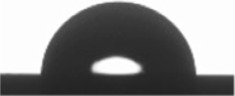	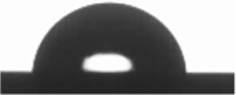
NbOx	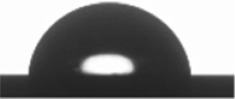	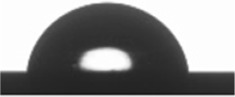	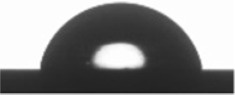	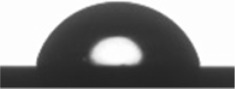	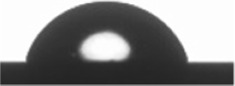	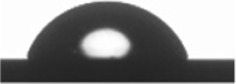
ZrOx	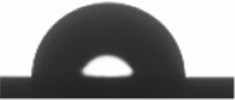	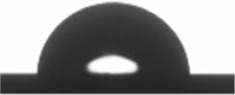	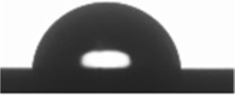	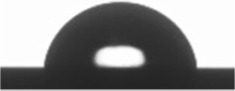	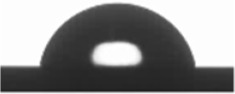	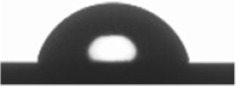

**Table 4:** Water Contact Angle (WCA) and Surface Free Energy (SFE) of uncoated and coated silicon surfaces with metal oxides thin films.

Samples	WCA (°)	Surface Free Energy (mN/m)			γ_p_/(γ_p_ +γ_d)_ (%)
		γ_d/p_	γ_d_	γ_p_	
Si	79.8 ± 0.9 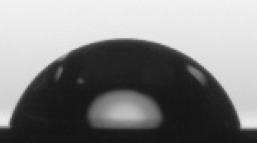	30.0 ± 0.1	21.2 ± 0.4	8.8 ± 0.5	29.3
TiOx	87.6 ± 0.5 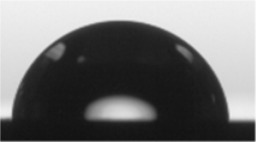	26.2 ± 0.1	21.2 ± 0.3	5.1 ± 0.2	19.4
TaOx	88.3 ± 0.9 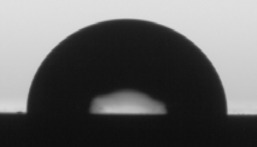	22.6 ± 0.2	14.9 ± 0.3	7.7 ± 0.5	34.0
NbOx	86.3 ± 1.2 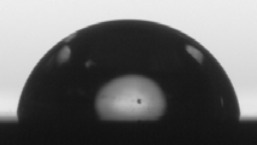	26.1 ± 0.1	19.5 ± 0.4	6.7 ± 0.5	25.7
ZrOx	89.2 ± 0.9 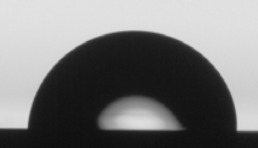	23.7 ± 0.1	18.0 ± 0.4	5.7 ± 0.4	24.0

γ_d/p,_ total SFE; γ_d_, SFE dispersive component; γ_p,_ SFE polar component; γ_p_/(γ_p_ + γ_d_), polarity factor.

## Data Availability

The data presented in this study are available on request from the corresponding author. The data are not publicly available due to ongoing research.

## Supplementary Materials

The following supporting information can be downloaded at: https://www.mdpi.com/article/10.3390/ma15155240/s1, Figures S1 and S2: Bone-marrow derived mesenchymal stem cells characterization and Figures S3 and S4: Immunofluorescence assays controls.
