# Supplementary material for: Evaluation of the Biocompatibility and Osteogenic Properties of Metal Oxide Coatings Applied by Magnetron Sputtering as Potential Biofunctional Surface Modifications for Orthopedic Implants
Source: Materials (Basel). 2022 Jul 29;15(15):5240. doi: 10.3390/ma15155240 (PMC9369574; doi:10.3390/ma15155240)
Supplement: Supplementary file 1 [file materials-15-05240-s001.zip › materials-1788699-supplementary.pdf]

Supplementary Information

Bone-marrow derived mesenchymal stem cells characterization

Even though BM-MSC in passage 1 were obtained with a quality control certificate from ATCC®, their expression of positive and negative mesenchymal stem cells markers and its differentiation capacity towards chondrogenic, adipogenic and osteogenic lineages were evaluated at passage 6-8 by flow cytometry and histological staining. The expression of positive, CD90-FITC (BD-555595), CD105-PE (BD-560839), CD73-APC (BD-560847), and negative CD45-FITC (BD-555482), CD34-PE(BD-550761), and HLA-DR-APC (BD-559866) MSC surface markers (BD, New Jersey, USA) from BM-MSC in passage 6 was measured in a flow cytometer FACSCalibur™ (BD, New Jersey, USA) using the Cell Quest Pro V.5.2.1.™ and the FlowJo V.10.8.1™ software. To evaluate the differentiation capacity of the BM-MSC towards the chondrogenic, adipogenic and osteogenic lineages, BM-MSC cells were incubated in specific differentiation-inducing culture media. For chondrogenic differentiation, the StemPro® Osteocyte/Chondrocyte Differentiation Basal Medium A10069-01 (Gibco™, New York, USA) supplemented with the StemPro® Chondrogenesis Supplement A10064-01 (Gibco™, New York, USA) and 1% antibiotic-antimycotic (Gibco™, New York, USA) was used. In the case of osteogenic differentiation, the same basal medium and antibiotic-antimycotic as described for chondrogenic differentiation were used but supplemented with the StemPro® Osteogenesis Supplement A10066-01 (Gibco™, New York, USA). To induce adipogenic differentiation, the StemPro® Adipogenesis Differentiation Basal Medium A10410-01 (Gibco™, New York, USA) supplemented with the StemPro® Adipogenesis Supplement A10065-01 (Gibco™, New York, USA) and 1% antibiotic-antimycotic (Gibco™, New York, USA) was used. Cell culture was carried out according to the differentiation-inducing media manufacturer protocols. Independent cell cultures were incubated at 37 °C, 5% CO_2 for 14, 17 and 12 days in chondrogenic, osteogenic and adipogenic differentiation medium, respectively. The time choice for evaluation of the chondrogenic, osteogenic and adipogenic cell differentiation was chosen according to the differentiation-inducing media manufacturer´ protocols.

After corresponding incubation time, cells were fixed with paraformaldehyde 4% (PFA; Sigma Aldrich, USA) for 15 min at room temperature (RT) and corresponding histological staining was performed, depending on the specific differentiation-inducing media used. Alcian blue staining was used to identify differentiation towards the chondrogenic lineage; briefly, fixed cells were successively rinsed with Phosphate Buffer Saline (PBS) 1X (Gibco™, New York, USA), distilled water and HCl 0.1 M (Merck, Darmstadt, Germany), incubated overnight in 1% filtered solution of alcian blue (Sigma-Aldrich, Missouri, USA) at pH 1, and finally rinsed with HCl 0.1 M and distilled water. Micrographs were acquired at 5X and 10X magnifications. For characterization of differentiation towards the osteogenic lineage, alizarin red staining was used; briefly, fixed cells were successively rinsed with (PBS 1X and distilled water, incubated in filtered 2% solution of alizarin red (72-48-0, Sigma-Aldrich, Missouri, USA) for 15 min at RT in the darkness, and finally rinsed with distilled water. Micrographs were acquired at 20X and 32X magnifications. To identify differentiation towards the adipogenic lineage, oil red staining was used; briefly, fixed cells were rinsed twice with PBS 1X and distilled water, dehydrated

at 60 °C for 7 min with 85% propylene glycol, incubated at 60 °C for 7 min in saturated solution of oil red O (26125; Merck Millipore, Massachusetts, USA) in propylene glycol, and rinsed successively with propylene glycol 85% and distilled water. Finally, cells nuclei were counterstained with Mayer´s hematoxylin (Biopack®, Buenos Aires, Argentina), and micrographs were acquired at 20X and 32X magnifications. All micrographs were acquired in an Axiovert 25 Microscope (Carl Zeiss, Germany)

Characterization of BM-MSC in passage 6 was carried out evaluating by flow cytometry the expression of the main positive and negative mesenchymal stem cells surface markers as indicated by the International Society of Cell Therapy; as well as its capacity to differentiate towards the chondrogenic, adipogenic, and osteogenic lineages upon incubation with specific differentiation-inducing media. Figure S1 shows BM-MSC cell positive expression of the surface mesenchymal stem cell markers CD90, CD105, and CD73, and lack of expression of the hematopoietic stem cell markers CD45, CD34, and HLA.

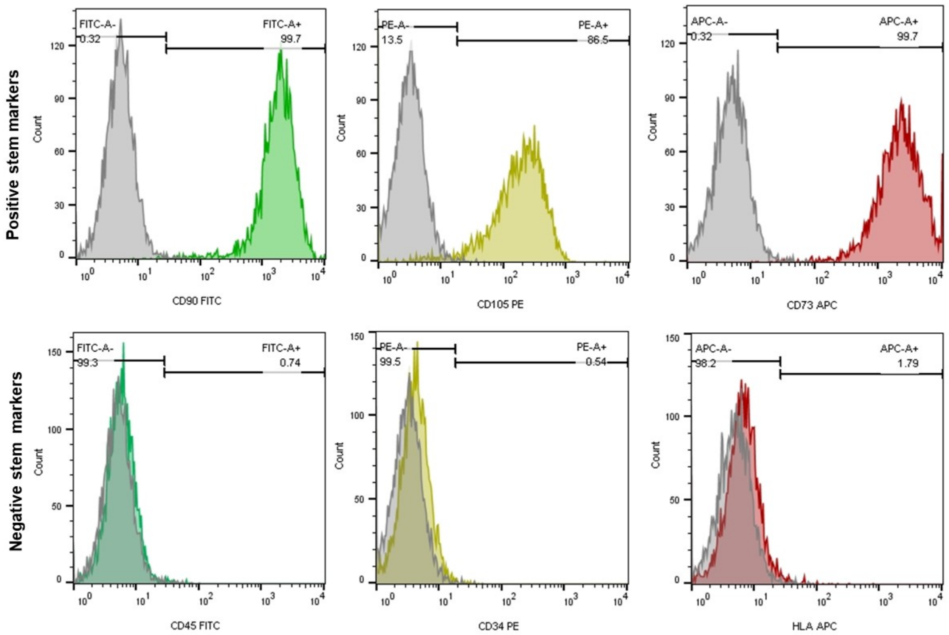

Figure S1. Flow cytometry characterization of BM-MSC cells (ATCC, PCS-500-012) in passage 6. Positive expression of surface stem cell markers CD90, CD105, and CD73; and lack of expression of hematopoietic stem cell markers CD45, CD34, and HLA.

In Figure S2(a), it is observed the fibroblast-like cell morphology of BM-MSC (passage 8) incubated in supplemented mesenchymal stem cell basal medium. Figure S2(b) shows BM-MSC cells after 14 days of culture in chondrogenic-inducing medium. The cell pellet was positively stained with alcian blue, indicating synthesis of proteoglycans, and consequently chondrogenic differentiation. Figure S2(c) shows BM-MSC after 17 days of incubation in osteogenic-inducing differentiation medium. Positive staining with alizarin red exhibited the formation of calcium deposits, indicative of osteogenic differentiation. Figure S2(d) shows BM-MSC after 12 days of incubation in adipogenic-inducing differentiation medium. Lipid vesicles can be clearly observed stained with oil red dye, demonstrating adipogenic differentiation. It was confirmed that BM-MSC cells used in the

present study have the main characteristics of mesenchymal stem cells up to passage 6, according to the International Society of Cell Therapy.

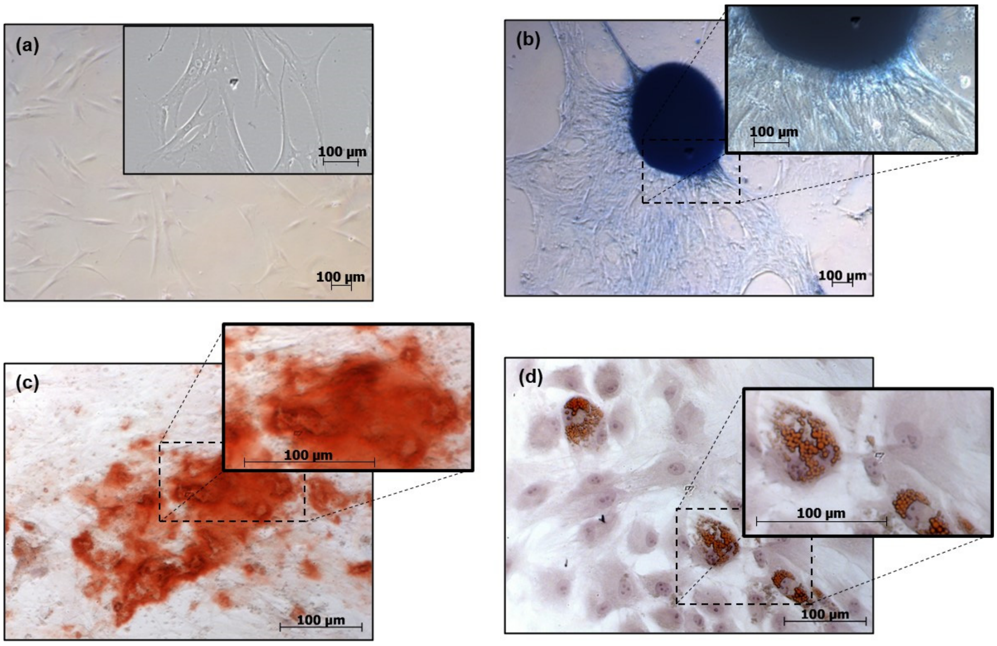

Figure S2. (a) Adherent fibroblast-like cell morphology of BM-MSC after 72 h of culture in supplemented mesenchymal stem cell basal medium. (b) Chondrogenic differentiation of BM-MSC after 14 days of culture in StemPro® Chondrogenesis Differentiation kit medium; alcian blue staining was used to identify proteoglycans expression. (c) Osteogenic differentiation of BM-MSC after 17 days of culture in StemPro® Osteogenesis Differentiation kit medium; alizarin red staining was used to identify calcium deposits. (d) Adipogenic differentiation of BM-MSC after 12 days of culture in StemPro® Adipogenesis Differentiation kit medium; oil red staining was used to stain lipid vesicles.

Immunofluorescence assay controls

In Figure S3, it can be corroborated the specificity of the secondary antibodies for the primary antibodies used in the IF technique; there was no visible green marking when primary antibody was absent.

In Figure S4(a), S4(e) and S4(i), it is observed in green the positive expression for RUNX2, OP and OC, which is characteristic of the hFOB. It has been reported that FBS present in culture media induces osteogenic differentiation in MSC (Ansari et al., 2022.). BM-MSC were seeded with different culture media in order to choose the medium inducing the lower expression of osteogenic markers. Figures S4(b), S4(f), and S4(j) show BM-MSC incubated with DMEM/F-12 supplemented with 2% FBS; figures S4(c), S4(g), and S4(k) show the cells incubated with DMEM/F-12 supplemented with 10% FBS; and Figures S4(d), S4(h), and S4(l) how cells incubated with supplemented mesenchymal stem cell basal medium. For further osteoinduction experiments in the present study, FBS was not to completely

removed from the culture medium due to the long incubation times used for IF assays. Ofut of the three media used, a higher expression of the osteogenic markers tested was observed in cells incubated in DMEM/F-12 supplemented with 10% FBS. Regarding the two-remaining media, a minimal expression of osteogenic markers was observed in both. Cells incubated in supplemented Mesenchymal Stem Basal Medium allowed us to corroborate that BM-MSC intrinsically express a certain amount of the proteins involved in the osteogenic differentiation process, but this amount was not comparable with the expression observed in the hFOB. Also, the supplemented DMEMF-12 with 10% FBS cannot be used to test if the coated silicon surfaces induce the osteogenic differentiation in BM-MSC. Thus, BM-MSC seeded on the coated silicon surfaces were incubated with DMEM/F-12 supplemented with 2% FBS.

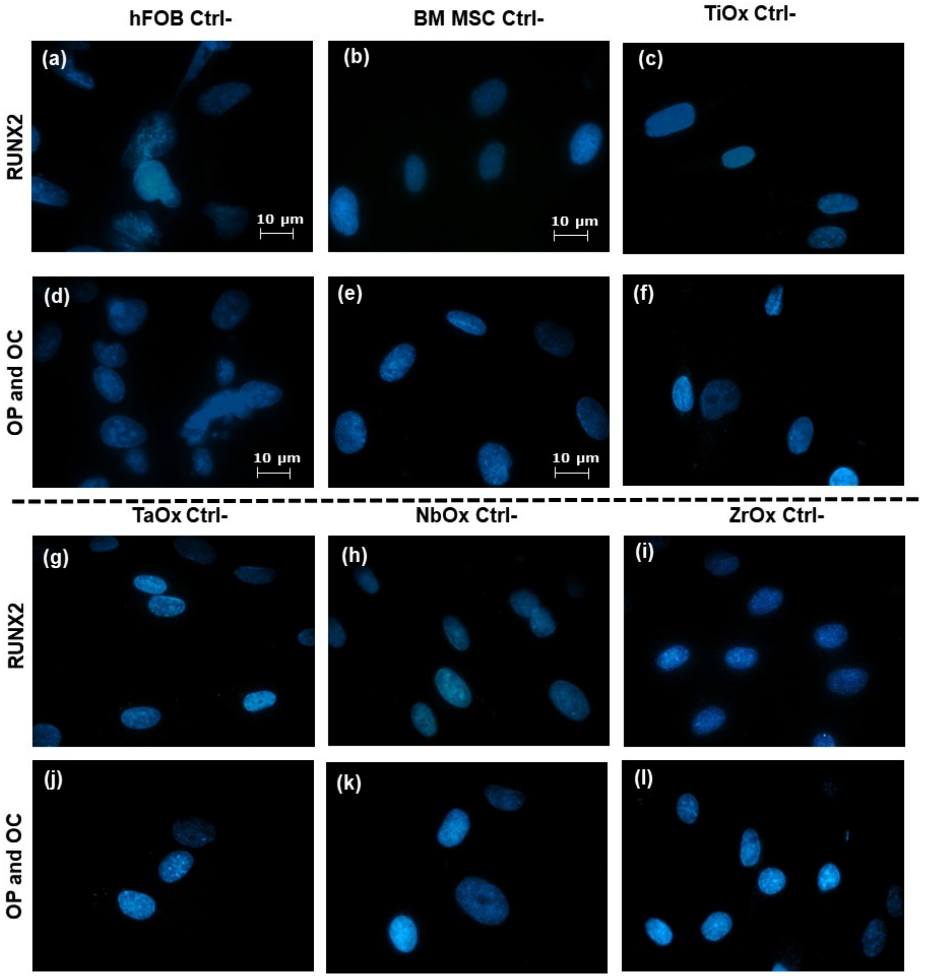

Figure S3. Negative controls of the Immunofluorescence (IF) technique, corresponding to BM-MSC seeded on coverslips and on the oxide coated silicon surfaces. For the secondary antibody specificity evaluation, in these controls the primary antibody was not added, only the secondary antibody was used. In blue, the cell nuclei stained with Invitrogen Hoechst 33342. (a,d) hFOB; (b, c, e, f, g, h, i, j, k, l) BM-MSC; (a, b, c, g, h, i). For RUNX, the secondary antibody was Donkey Anti-Mouse ab150105, Alexa Fluor® 488. (d, e, f, j, k, l). For OP and OC, the

secondary antibody was Donkey Anti-Rabbit Alexa Fluor® 488. hFOB: Osteoblasts. BM MSC: Bone marrow-derived mesenchymal stem cell line.

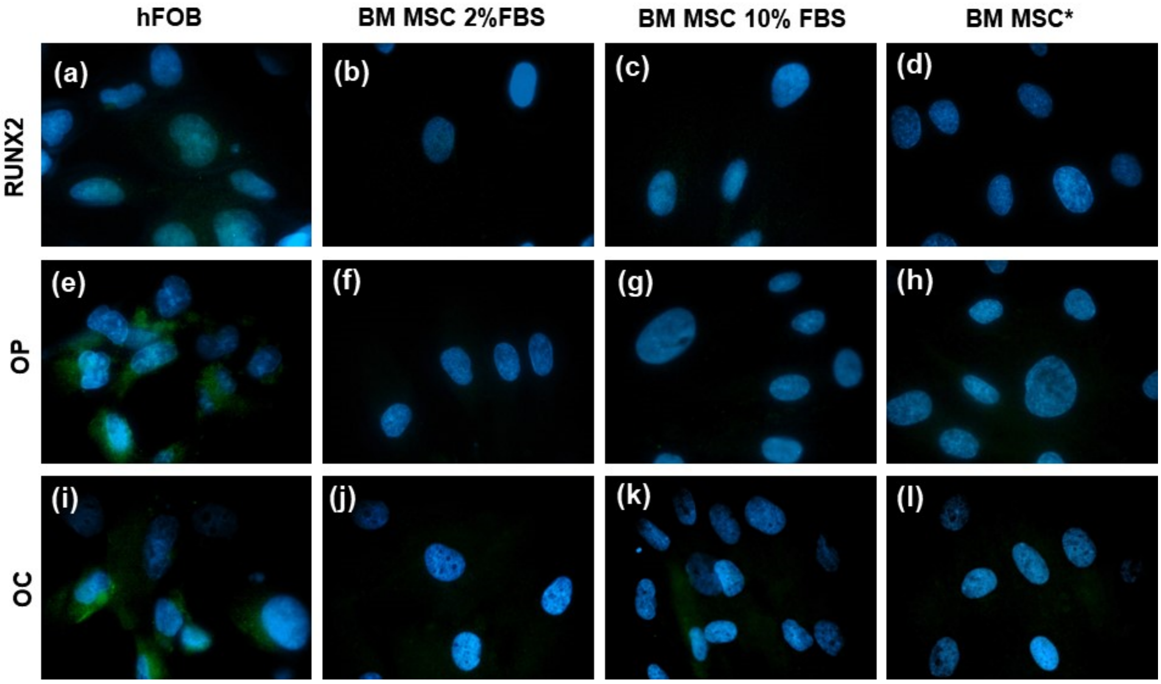

Figure S4. Cell expression controls. Cells were seeded on coverslips. Incubation conditions were the same as for BM-MSC seeded on the coated silicon surfaces, as well as the IF technique. (a, e, i) hFOB, positive control of the expression of RUNX2, OP and OC. (b, c, d, f, g, h, j, k, l). BM-MSC, negative control of the expression of RUNX2, OP and OC. In green, a positive expression of the corresponding osteogenic marker is observed, while in blue, the cell nuclei stained with Invitrogen Hoechst 33342. hFOB: Osteoblasts. BM MSC: Bone marrow-derived mesenchymal stem cell line. BM MSC 2% FBS: Cells incubated in DMEM/F-12 with 2% FBS. BM MSC 10% FBS: Cells incubated in DMEM/F-12 with 10% FBS. BM MSC *: Cells incubated in supplemented Mesenchymal Stem Cell Basal Medium.
